# Pharmacological treatment with inhibitors of nuclear export enhances the antitumor activity of docetaxel in human prostate cancer

**DOI:** 10.18632/oncotarget.22760

**Published:** 2017-11-30

**Authors:** Giovanni Luca Gravina, Andrea Mancini, Alessandro Colapietro, Francesco Marampon, Roberta Sferra, Simona Pompili, Leda Assunta Biordi, Roberto Iorio, Vincenzo Flati, Christian Argueta, Yosef Landesman, Michael Kauffman, Sharon Shacham, Claudio Festuccia

**Affiliations:** ^1^ Department of Biotechnological and Applied Clinical Sciences, Laboratory of Radiobiology, University of L’Aquila, L’Aquila, Italy; ^2^ Department of Biotechnological and Applied Clinical Sciences, Division of Radiotherapy, University of L’Aquila, L’Aquila, Italy; ^3^ Department of Biotechnological and Applied Clinical Sciences, Division of Human Anatomy, University of L’Aquila, L’Aquila, Italy; ^4^ Department of Biotechnological and Applied Clinical Sciences, Division of Molecular Pathology, University of L’Aquila, L’Aquila, Italy; ^5^ Department of Biotechnological and Applied Clinical Sciences, Division of Applied Biology, University of L’Aquila, L’Aquila, Italy; ^6^ Karyopharm Therapeutics, Newton, MA, USA

**Keywords:** castration resistant prostate cancer (CrPCa), chromosome region maintenance (CRM-1), exportin-1 (XPO-1), selective inhibitors of nuclear export (SINE), docetaxel (DTX)

## Abstract

**Background and aims:**

Docetaxel (DTX) modestly increases patient survival of metastatic castration-resistant prostate cancer (mCRPC) due to insurgence of pharmacological resistance. Deregulation of Chromosome Region Maintenance (CRM-1)/ exportin-1 (XPO-1)-mediated nuclear export may play a crucial role in this phenomenon.

**Material and methods:**

Here, we evaluated the effects of two Selective Inhibitor of Nuclear Export (SINE) compounds, selinexor (KPT-330) and KPT-251, in association with DTX by using 22rv1, PC3 and DU145 cell lines with their. DTX resistant derivatives.

**Results and conclusions:**

We show that DTX resistance may involve overexpression of β-III tubulin (TUBB3) and P-glycoprotein as well as increased cytoplasmic accumulation of Foxo3a. Increased levels of XPO-1 were also observed in DTX resistant cells suggesting that SINE compounds may modulate DTX effectiveness in sensitive cells as well as restore the sensitivity to DTX in resistant ones. Pretreatment with SINE compounds, indeed, sensitized to DTX through increased tumor shrinkage and apoptosis by preventing DTX-induced cell cycle arrest. Basally SINE compounds induce FOXO3a activation and nuclear accumulation increasing the expression of FOXO-responsive genes including p21, p27 and Bim causing cell cycle arrest. SINE compounds-catenin and survivin supporting apoptosis. βdown-regulated Cyclin D1, c-myc, Nuclear sequestration of p-Foxo3a was able to reduce ABCB1 and TUBB3 H2AX levels, prolonged γ expression. Selinexor treatment increased DTX-mediated double strand breaks (DSB), and reduced the levels of DNA repairing proteins including DNA PKc and Topo2A. Our results provide supportive evidence for the therapeutic use of SINE compounds in combination with DTX suggesting their clinical use in mCRPC patients.

## INTRODUCTION

Prostate cancer (PCa) is the second leading cause of cancer-related death in males in industrialized western countries and represents a growing problem worldwide [1]. Hormone refractory/castration resistant PCa (CRPC) usually develops after an initially therapeutically efficacious treatment with anti-hormonal compounds [2]. CRPC is also associated with resistance to conventional chemotherapies. Docetaxel (DTX), a first line treatment for CRPC, confers survival advantages of approximately 2 months for patients [3, 4] with low overall survival benefit. Indeed, most patients with CRPC relapse and become resistant to DTX therapy [4–7]. Despite the high prevalence of DTX-refractory disease, little is known about the tumor biology of the DTX-resistant residual tumor cells compared with primary tumor cells. Gene expression profiling of androgen independent prostate cancer cells demonstrates complex mechanisms mediating resistance to DTX [10]. In addition, ABC drug transporter family proteins [8] and clusterin [7, 9], have been proposed to play a role in resistance. Accumulating evidence suggests that more aggressive disease develops after DTX-resistance [11, 12] with recruitment and activation of cancer stem [13, 14]. In breast cancer, it was recently shown that residual cancer cells increased mammosphere formation efficacy when compared to primary cancers [9]. Thus, the tumorigenic potential of residual/DTX-resistant (DTXR) cancer cells may be enhanced if the cancer cells were not eradicated by chemotherapy.

It has been previously observed that tumorigenic potential was increased in DTXR residual prostate cancer cell lines when compared to parental cells [15]. Tumorigenic potential in DTXR PCa could be conferred by oncogenic c-Myc, which was stabilized by constitutively activated ERK1/2 in resistant cells. Constitutively activated ERK1/2 was maintained by CXCR4, which was also upregulated in resistant cells. It is now commonly accepted that constitutive activation of the CXCR4, ERK1/2, and c-Myc signaling loop plays a major role in prostate tumorigenesis [15, 16].

The nucleo-cytoplasmic shuttling of proteins and RNAs is a dynamic process that is regulated by the exportin family of proteins. Exportin-1 (XPO-1), also known as chromosomal region maintenance 1 (CRM1), shuttles about 220 proteins from the nucleus to the cytoplasm [17, 18] and is the sole nuclear exporter of several tumor suppressor (TSP) and growth regulatory (GRP) proteins, such as p53 and p73 [19, 20], p21 [21], survivin [22, 23], cyclin D1 [23, 24], Rb1 [25], apc [26], bcr-abl [27], FOXO [28], p27 [29] and STAT3 [30]. Physiologically, export of these proteins prevents unnecessary activity in the absence of DNA injury and other oncogenic activities [31–33]. In tumor cells, however, the export of these proteins inhibits their activity thus promoting tumorigenesis. Many hematologic and solid tumors have elevated XPO-1 levels [34–37] with a strong correlation between increased XPO-1 expression and poor prognosis, as demonstrated in osteosarcoma [38], pancreatic [39], lung [35] and ovarian cancers [34].

PI3K/Akt/mTOR/GSK3β signaling has been shown to increase XPO-1 dependent nuclear export of TSPs, whereas its inhibition [40–43] as well as that of XPO-1 [22, 23] reduces this turnover/recycling. In contrast, PTEN is known to antagonize oncogenic PI3K/Akt signaling. The expression of PTEN, a cargo of XPO-1, is repressed or lost in PCa [45, 46]. It has been also described that the shuttling of cyclin D1 (CCND1 [23], responsible for the progression of cell cycle and regulating the G1/S-phase transition of proliferating cells [44]), from the nucleus to the cytoplasm is regulated by XPO1. Inhibition of XPO-1 and thus the nuclear export of cyclin D1 may activate caspase activity and apoptotic machinery. Similarly, surviving, when localized in nucleus, is mainly involved in spindle monitoring at mitosis, whereas cytoplasmic/mitochondrial survivin counteracts pro-apoptotic signals by preventing caspase-9 and caspase-3 activation, thus XPO-1 inhibition and the nuclear enrichment of survivin would inhibit the anti-apoptotic activity of survivin [22].

A recently developed series of slowly reversible orally available Selective Inhibitor of Nuclear Export (SINE) compounds bind to XPO-1 and inhibit XPO-1 mediated nuclear export [for review see 40]. SINE compounds are actively being evaluated in human [46, 47] and animal clinical trials [48, 49]. In our study, we investigated whether two SINE molecules (KPT-251 and KPT-330/ selinexor) would also be effective against CRPC cells in combination with DTX. We hypothesize that elevated XPO-1 expression is associated with DTXR, as described in a previous report [50], and that the inhibition of this cargo protein may sensitize to DTX in both naive and resistant clones. Here, we show that XPO-1 inhibition using KPT-251 and selinexor enhances the sensitivity to DTX and conclude that the XPO-1 mediated shuttling of nuclear proteins might play an important role in inducing resistance to chemotherapeutic drugs in PCa cells. Together this data suggests that targeting XPO-1 might be a promising strategy for enhancing sensitivity to chemotherapy in CRPC.

## RESULTS

### Effects of docetaxel in DTX sensitive PC3, 22rv1 and DU145 cell lines

**Figure 1:**
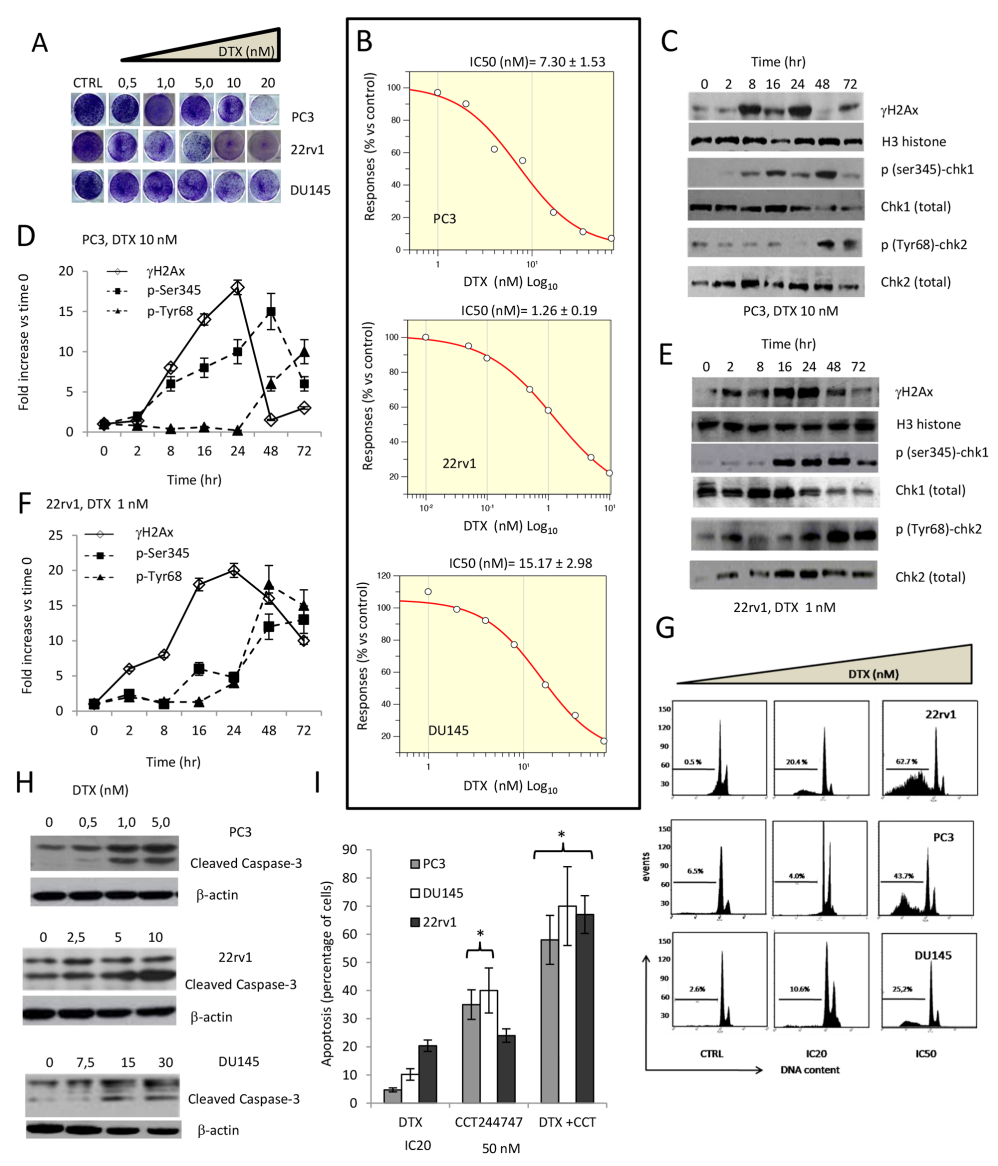
Effects of docetaxel (DTX) in DTX sensitive (DTXS) PC3, 22rv1 and DU145 cell lines **(A)** Plate image representation of crystal violet stained PC3, 22rv1 and DU145 cells cultured in 24 well/plates with different doses of DTX (0-20 nM). **(B)** Proliferation curve generated with Graftit software for PC3 (IC50=7.21 nM), 22rv1 (IC50=1.26 nM) and for DU145 cells (IC50=15.17 nM). **(C)** DTX (10.0 nM) induces time-dependent DNA damage response (DDR) characterized by γ-H2AX phosphorylation and Chk1/2 kinase activation in PC3 cells. **(D)** The levels of γH2AX, Chk1 and Chk2 were normalized by using H3 histone as housekeeping nuclear protein, whereas those of phosphorylated forms of Chk1 (p-Ser345) and Chk2 (p-Tyr68) with total expression of Chk1 and chl2, respectively. Normalizing expression levels were plotted in the time for PC3 cells. **(E)** DTX (1.0 nM) induces time-dependent DNA damage response (DDR) characterized by γ-H2AX phosphorylation and Chk1/2 kinase activation in 22rv1 cells. **(F)** The levels of γH2AX, Chk1 and Chk2 were normalized by using H3 histone as housekeeping nuclear protein, whereas those of phosphorylated forms of Chk1 (p-Ser345) and Chk2 (p-Tyr68) with total expression of Chk1 and chl2, respectively. Normalizing expression levels were plotted in the time for 22rv1 cells. **(G)** FACS analyses for apoptotic rate in CTRL and DTX treated cells at IC20 and IC50 values. IC20 values were 0.5 nM, 5.8 nM and 10 nM for 22rv1, PC3 and DU145, respectively **(H)** caspase 3 activation /cleavage. **(I)** Addition of 50 nM Chk1 inhibitor CCT244747 start and accelerate the program of cell death in PC3, 22rv1 and DU145 cells. Each lane of western blots was loaded with 100 μg of proteins. Graphical data derived from three different western blot analysis performed on different cell extracts. Data presented as mean ±Error Standard (ES). Data for γH2AX are statistically significant at all considered times whereas p(SER235)-Chk1 and p(Tyr68)-Chk2 levels were significant starting from 16 and 24 hour, respectively (p<0.001).

First, we analyzed the effects of DTX on PC3, DU145 and 22rv1 cell lines. In Figure 1A we show the plate image representation of crystal violet stained PC3, 22rv1 and DU145 cells cultured in 24 well/plates with different doses of DTX (0-20 nM). Proliferation of these cells was inhibited by DTX (Figure 1B) with IC_50_ values of 7.20 nM in p53 null PC3 cells, 1.26 nM in p53 wild type 22rv1 cells, and 16.17 nM PTEN mutant and inactive DU145 cells. It is well known that DTX is able to induce replication stress promoting DSBs and leading to activation of multiple molecular pathways regulating DNA repair, including ATM-Chk2-p53 (22rv1 cell line), ATR-Chk1 (PC3 and DU145 cell lines) and PARP signaling. In addition we observed that DTX increased the levels γH2AX and phosphorylation of Chk1/2, which are indicative of DNA damage and kinase activity, respectively. In Figure 1C and 1E we show western blotting evaluation on molecular arrangement induced by 10 nM in p53 mutated PC3 cells (1C) and 1 nM in p53 wild type 22rv1 cells (1E). DTX doses close to the IC_50_ values were chosen for both cell lines. The levels of γH2AX, Chk1 and Chk2 were normalized using histone H3, whereas phosphorylated forms of Chk1 (p-Ser345) and Chk2 (p-Tyr68) were normalized to total expression of Chk1 and Chk2, respectively. From a first analysis of western blotting of PC3 and 22rv1 cells treated with DTX, γH2AX reached maximal levels at 24 hours then dropped to reach sub-baseline values. In 22rv1 cells (Figure 1F) the increase of γH2AX was more gradual without a clear return to basal values. This data suggests that 22rv1 cells were actively trying to repair DNA but could not. As a result, they again activated DDR, as evidenced by high γH2AX levels. PC3 cells seem to have completed, instead, the DNA-repair and could resume their cell proliferation after an initial cytostasis. The levels of Chk1 and Chk2 were significantly reduced in the time indicating possible shuttling in the cytoplasm and degradation. The reduction of Chk1 levels was higher when compared to those observed for Chk2. The normalized levels of the phosphorylated nuclear isoforms of Chk1 and Chk2 (Figure 1D, 1F) were increased in the time especially in 22rv1 cells. Chk1 and Chk2 activation was consistent with that observed for γΗ2AX. The levels p-(ser345)Chk1 increased in PC3 and 22rv1 (Figure 1D, 1F) following the increment of γΗ2Ax levels and were maximal at 48 hours although γΗ2Ax expression levels dropped in PC3 cells (Figure 1D). Levels of p-Chk2(Tyr68) increased both in PC3 and 22rv1 cells. Chk1 and Chk2 activation levels are different in PC3 and 22rv1cells and this suggests differences in the DTX-mediated cell apoptosis (Figure 1G) and caspase 3 activation (Figure 1H). In PC3 cells (in the absence of p53) DNA damage signals a replicative block in S and G2/M cell cycle phase. In 22rv1 (in the presence of p53) a further replicative block may be observed in the passage from G1 to S. In the Figure 1G we show FACS determinations demonstrating a bigger G2/M cell peak, corresponding to a cell cycle arrest in this phase, in PC3 cells whereas a reduction of this peak observed in 22rv1 cells, was indicative of G0/G1 cell cycle arrest and this was in agreement with several literature data. DU145 cells, with mutant and not functional p53, follow the trend of PC3 cells also if the G2/M peak in the administration of the IC50 dose of DTX seemed to be reduced compared to that observed at IC20 dose. The block of G2/M was associated also with increased subG1 apoptotic cells ranged between 25% (PC3 cells) and 67% (22rv1 cells). In addition we demonstrate a critical role of Chk1 in the protection to cell death. The addition of 50 nM Chk1 inhibitor CCT244747 (Figure 1I), indeed, breaching down Chk1 activity allows to start and thus accelerate the program of cell death.

**Figure 2:**
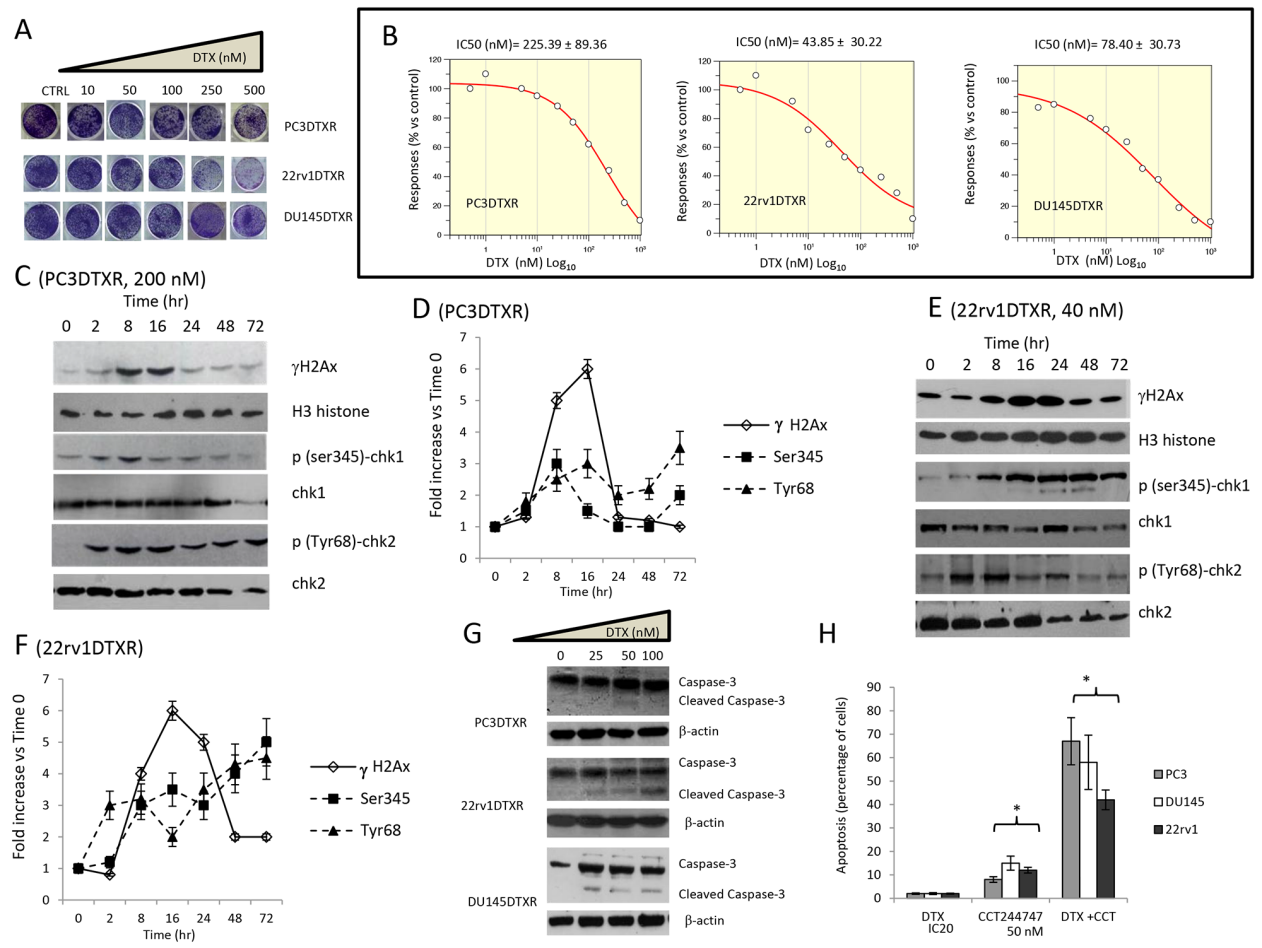
Effects of docetaxel (DTX) in DTX resistant (DTXR) PC3, 22rv1 and DU145 cell lines **(A)** Plate image representation of crystal violet stained PC3DTXR, 22rv1DTXR and DU145DTXR cells cultured in 24 well/plates with different doses of DTX (0-500 nM). **(B)** Proliferation curve generated with Graftit software for PC3DTXR (IC50=225.39 nM), 22rv1 (IC50=43.85 nM) and for DU145 cells (IC50=78.40 nM). **(C)** DTX (200 nM) induces time-dependent DNA damage response (DDR) characterized by γ-H2AX phosphorylation and Chk1/2 kinase activation in PC3DTXR cells. **(D)** The levels of γH2AX, Chk1 and Chk2 were normalized by using H3 histone as housekeeping nuclear protein, whereas those of phosphorylated forms of Chk1 (p-Ser345) and Chk2 (p-Tyr68) with total expression of Chk1 and chl2, respectively. Normalizing expression levels were plotted in the time for PC3DTXR cells. **(E)** DTX (40 nM) induces time-dependent DNA damage response (DDR) characterized by γ-H2AX phosphorylation and Chk1/2 kinase activation in 22rv1DTXR cells. **(F)** The levels of γH2AX, Chk1 and Chk2 were normalized by using H3 histone as housekeeping nuclear protein, whereas those of phosphorylated forms of Chk1 (p-Ser345) and Chk2 (p-Tyr68) with total expression of Chk1 and chl2, respectively. Normalizing expression levels were plotted in the time for 22rv1DTXR cells. **(G)** caspase 3 activation /cleavage. **(H)** Addition of 50 nM Chk1 inhibitor CCT244747 start and accelerate the program of cell death in PC3DTXR, 22rv1DTXR and DU145DTXR cells. Each lane of western blots was loaded with 100 μg of proteins. Graphical data derived from three different western blot analysis performed on different cell extracts. Data presented as mean ±Error Standard (ES). In PC3DTXR cells, data for γH2AX are statistically significant starting to 2 hours and until 24 hours; p(SER235)-Chk1 was statistically significant at 8 hour and at 72 hour whereas only p(Tyr68)-Chk2 levels significantly followed a time-dependent increase (p<0.001). In 22rv1DTXR γH2AX, p(Ser235)-Chk1 and p(Tyr68)-Chk2 levels followed a statistically significant time-dependent increase (p<0.001).

Next, we confirmed that DTX resistant cell derivatives were effectively resistant to this compound. In Figure 2A we show plate image representations of crystal violet stained DU145DTXR, 22rv1DTXR and PC3DTXR cells grown in 24 well plates cultured with different doses of DTX (0-500 nM) and having significant higher IC_50_ values (Figure 2B) to those observed for DTXS cell strains reaching 225 nM (PCE3DTXR), 44 nM (22rv1DTRX) and 78 nM (DU145DTXR). Also in DTXR cells, DTX at IC_50_ values for each cell lines induced DNA damage response (DDR) characterized by γH2AX phosphorylation and Chk1/2 kinase activation (Figure 2C in PC3DTXR and Figure 2E in 22rv1DTXR cells). Our data shows that γH2AX reached maximal levels at 8-16 hours both in PC3DTXR (Figure 2C, 2D) and 22rv1DTXR (Figure 2E, 2F) cells, then returned to baseline values at 48 and 72 hours suggesting that DNA damage was repaired. Chk1 kinase activation (p-Ser345) levels increased in a time-dependent manner in 22rv1 cells (Figure 2E, 2F) whereas reached maximal levels up to 16 hours in PC3 Figure 2C, 2D), then went down and remained at sub-baseline values. The levels of p-Tyr68 Chk2 remained elevated until the 8 hour time point (Figure 2D, 2F), then decreased at the 16 hour time point and reached maximum values at 48 and 72 hours. In both cell lines the levels of Chk1 and Chk2 were maintained high and the levels of γH2AX decreased, suggesting that DNA was not repaired. As expected, caspase-3 activity and apoptosis were not observed (Figure 2G) as the cells are DTXR. The protective role of Chk1 was further tested by using 50 nM CCT244747 (Figure 2H) showing synergistic effects in association with DTX.

**Figure 3:**
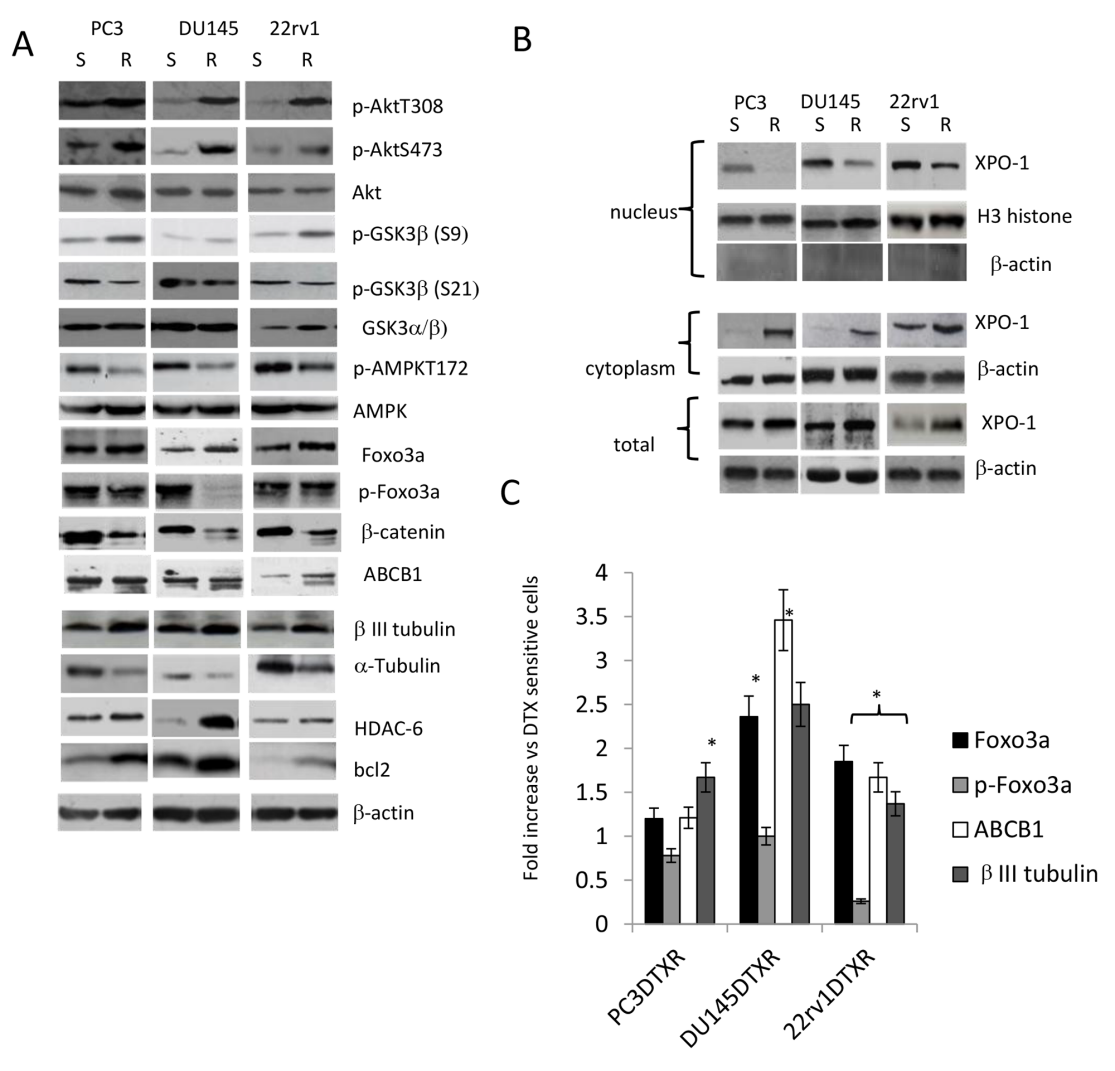
**(A)** Differences in molecular arrangements on DTX sensitive and resistant CRPC cell lines. **(B)** Nuclear and cytoplasmic expression of XPO1 in sensitive and resistant CRPC cell lines. Western blot for nuclear extracts were also blotted with anti β-actin antibody to verify the presence of cytosolic proteins. (B) Foxo3a, p-Foxo3a, ABCB1 and βIII tubulin levels in DTX resistant CRPC cells expressed as fold increase **(C)** vs DTX sensitive cells. Each lane was loaded with 100 mg of proteins. Graphs were made analyzing three different western blots. ^*^ p<0.001.

Next, we compared some molecular profiles in parental and resistant cell lines. Consistent with previous reports [57-59], we show that that DTXR cells had significant higher levels of βIII tubulin, Foxo3a and ABCB1 when compared to parental ones (Figure 3A, 3C). The expression of HDAC-6, which interacts with and deacetylates α-tubulin and microtubules *in vivo*, was also increased in resistant cells. The expression levels of α-tubulin were significantly lower in resistant cells when compared to parental ones (Figure 3A). 22rv1 DTXR cells showed no modulation of p-FOXO3a (S253), PC3 DTXR cells showed a slight reduction expression whereas DU145 cells showed a significant strong reduction of p-FOXO3a (S253) as shown in Figure 3A, 3C). DTX resistant cells showed also higher levels of p-AktT308 and p-AktS473. XPO-1 expression levels were higher in the cytoplasm when compared to nucleus of PCa cells and this difference was increased in DTXR respect to DTX sensitive (DTXS) cells. This was in agreement with our previous data showing that more aggressive PCa cells showed higher XPO1 levels and cytoplasmatic localization [23].

**Figure 4:**
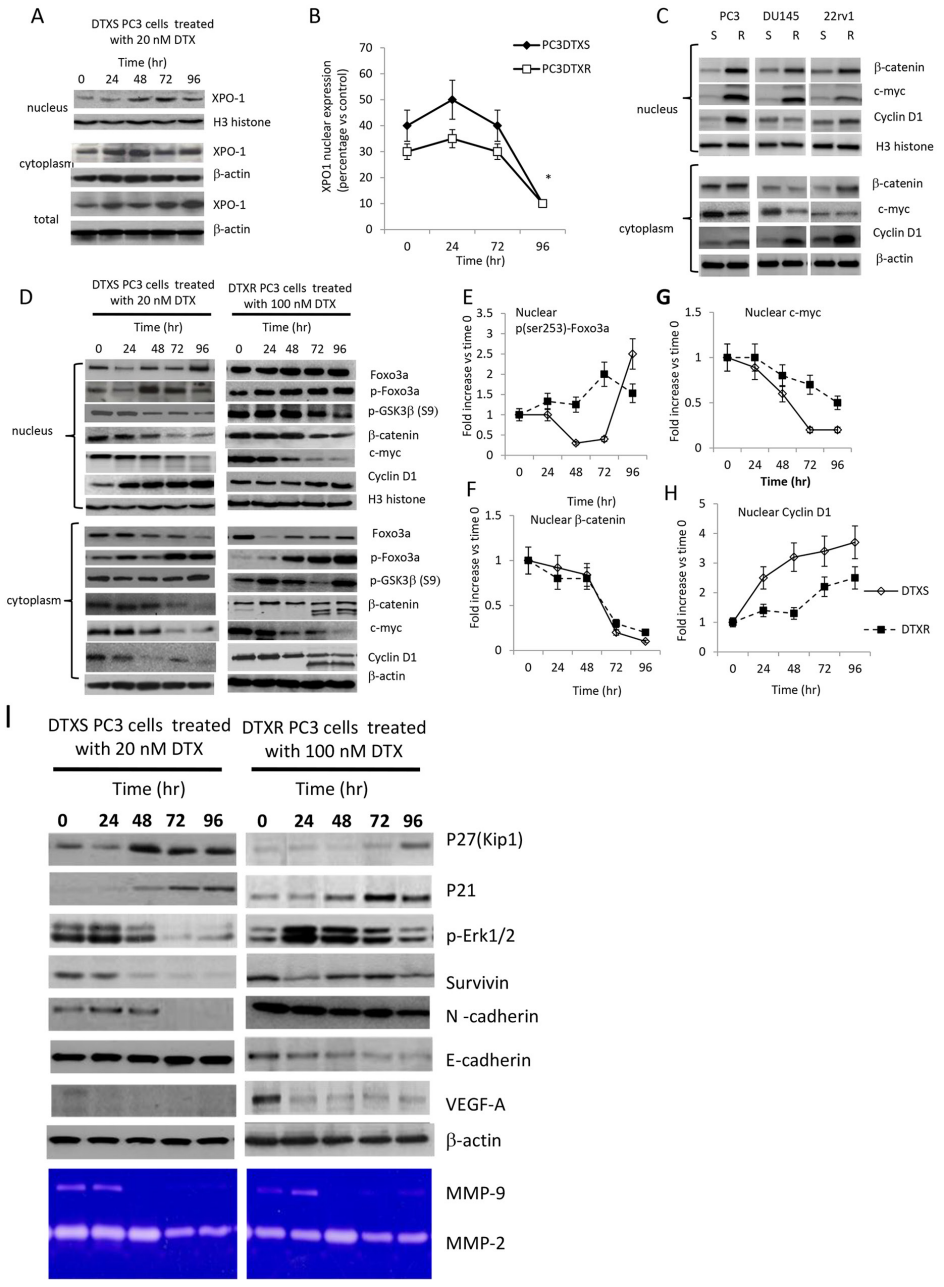
**(A)** Effects of 10 nM DTX in DTXS cells (time-dependent trend) on XPO1 expression in nuclear, cytoplasmic and total cell extracts. **(B)** Differences in the modulation of XPO1 expression, normalized vs Histone H3, during DTX administration in DTXS (DTX=10 nM) and DTXR (DTX=200 nM) PC3 cell strains. **(C)** Nuclear and cytoplasmatic Expression of β-catenin and its related targets (c-myc and cyclin D1) in DTX resistant and sensitive CRPC cells. **(D)** Modulation of nuclear and cytoplasmatic expression of GSK3β/Foxo3a/β-catenin and related target proteins (c-myc and cyclin D1) in DTXS and DTXR PC3 cell strains treated with 10 and 200 nM DTX, respectively. Immunoblots for p-Foxo3a, β-catenin, c-myc and cyclin D1 were normalized first versus the levels of Histone H3 and next on the levels at Time 0 (T0). Three western blots were analyzed and data expressed as mean of fold increases versus T0 ± Standard Error (SE). **(E)** Modulation of nuclear expression levels of p-Foxo3a in PC3DTXS and PC3DTXR cells; **(F)** modulation of nuclear expression levels of β-catenin in PC3DTXS and PC3DTXR cells; **(G)** modulation of nuclear expression levels of c-myc in PC3DTXS and PC3DTXR cells; **(H)** modulation of nuclear expression levels of cyclin D1 in PC3DTXS and PC3DTXR cells. **(I)** Western blot and zymographic evaluation of antiproliferative/cell cycle dependent (p21 and p27), EMT (Survivin, VEGF-A, N-cadherin and metalloproteinases) and Epithelial (E-cadherin) related targets.

In addition, we demonstrate that treatment with DTX was able to modulate the nuclear and cytoplasmatic levels of XPO1 (Figure 3B). In Figure 4A we show that administration of 20 nM DTX stimulated, in DTXS PC3 cells, an initial nuclear translocation of XPO1 at 24-48 hours (Figure 4A, 4B) and a late reduced nuclear expression. Similarly, DTXR PC3 cells treated with 100 nM DTX increased the levels of nuclear XPO1 at 24 hours which was reduced at very low extent at 72 and 96 hours (Figure 4B). A number of studies demonstrated a role for FOXO3a in tumor progression by promoting invasive migration of cancer cells [58, 59]. For example, FOXO3a promotes metastasis in the context of nucleus accumulation of β-catenin [59]. The finding suggests that nuclear FOXO3a and β-catenin may synergize to promote transcription of genes that are involved in scattering and metastasis. So, we first compared the nuclear and cytoplasmatic levels of β-catenin, c-myc and cyclin D1 (Figure 4C) and next the molecular re-arrangement induced by DTX on GSK3β/Foxo3a/β-catenin pathways both in DTXS and DTXR PC3 cells. We found that resistant strains showed high levels of oncogenic protein involved in the Foxo3a/β–catenin pathways. For example, cyclin D1 was equally distributed between cytoplasm and nucleus in both sensitive and resistant strains and was associated to elevated levels of β-catenin. Similarly c-myc was induced especially in the nuclear microenvironment and reduced in the cytoplasm of resistant DU145 cells.

After normalizations first versus total Foxo3a (for p-Foxo3a) or Histone H3 (for β-catenin, c-myc and cyclin D1) and next versus Time 0 (T0) we demonstrated that in the PC3DTXS cell strain, DTX administration significantly reduced the nuclear levels of p-Foxo3a (Figure 4D, 4E) up to 48 hours. Subsequently they went up to reach the maximum levels at 72 hours. In the PC3DTXR cell strain, instead, the nuclear p-Foxo3a levels grew in a time-dependent manner (Figure 4D, 4E). Parallely, cytoplasmatic expression of Foxo3a was progressively reduced in the time (Figure 4D) in both cell strains.

Several studies demonstrated a role for FOXO3a in tumor progression through the promotion of invasive migration of cancer cells [58, 59]. An important target of Foxo3a in the activation of this pathway is the β-catenin which undergoes continuous nuclear to cytoplasmic shuttling and vice versa, which regulates its function [59]. So, Foxo3a and β-catenin could synergize to promote transcription of genes that are involved in scattering and metastasis. Next we verified the nuclear and cytoplasmatic levels of β-catenin as well as the expression of some downstream targets of β-catenin (indicators of activity) such as c-myc, cyclin D1, cytokeratin 18, E-cadherin, N-cadherin and MMP-9. As expected higher β-catenin levels were found in PC3 DTXR cell strain when compared to PC3DTXS ones (Figure 4F) suggesting that β-catenin expression could be associated with drug resistance to DTX. So we verified if β-catenin levels were modified by DTX treatment in both cell strains. We observed that the levels of β-catenin were significantly reduced in the nucleus of both DTX sensitive or resistant treated PC3 cells with similar degree (Figure 4D, 4F). The analyses of the cytoplasmatic levels of this protein showed an increased proteolysis observed in PC3 DTXS cells at 72 and 96 hours of treatment with DTX (Figure 4D).

The expression of c-myc was significantly reduced in DTXS PC3 cells both in the nucleus and in the cytoplasm (Figure 4D). The comparison between DTX sensitive and resistant cells treated with DTX shows that c-myc undergoes a reduction of about 50% in resistant cells whereas that observed in sensitive cells was much more of 150% (Figure 4G). The levels of GSK3β were not significantly modified in DTXS PC3 cells whereas in DTXR PCX3 cells p-GSK3β levels progressively decreased after an early induction due probably to modifications in Akt activity. Nuclear accumulation was, instead, observed for Cyclin D1 (Figure 4H), a protein that must exit the nucleus in the transition from G2/M to G0/G1 of cell cycle. This justify further that PC3 are arrested in the G2/M cell cycle phase.

Expression of VEGF-A as well as N-cadherin, a marker for Epithelial-Mesenchymal Transition (EMT) are related to a β-catenin nuclear activity. So we verified if DTX sensitivity/resistance was associated to EMT phenotype. It has been well known that DTXR cells express higher levels of N-cadherin of DTXS cells. Similarly, VEGF-A secretion was higher in DTXR when compare to DTXS cells. Conversely, the levels of epithelial markers such as cytokeratin 18 (K18) or E-cadherin were significantly reduced in DTXR. Short-term administration of DTX was able to down-regulate N-cadherin and VEGF levels in DTXS cells whereas no changes were found in DTXR (Figure 4I) No significant modulation by DTX administration was found in zymographic analyses for MMP2 whereas MMP9 appear to be significantly down-regulated. Cell cycle dependent p21 and p27 proteins was upregulated indicating cell cycle arrest. The levels of Erk were up-regulated in DTXR cells whereas a significant down-modulation was observed in DTXS cells and this was in agreement with the higher effects of DTX in DTXS when compared to those observed in DTXR cells. DTXR cells result also protected by different intracellular signaling involving Akt and MAPK.

### XPO1 inhibition increases the sensitivity to DTX in PC3, DU145 and 22rv1 DTX sensitive cells and sensitizes DTXR PC3, 22rv1 and DU145 cells to DTX *in vitro*

The combination of selinexor with DTX demonstrated a significant reduction of tumor cell viability as evidenced by a dose-dependent chemotherapeutic agent induction of 50% cell death. For the Chou-Talay analyses of combination we examined PC3 and DU145 cells. Importantly, both cell lines are sensitive to selinexor (IC_50_ values for selinexor 380 and 105 nM for PC3 and DU145 cells, respectively) and DTX (7.2 and 15.2 nM for PC3 and DU145 cells, respectively). Treatments with DTX and selinexor in combination in CRPC cell lines was synergistic, additive or competitive depending on the concentrations used and the cell line studied.

**Figure 5:**
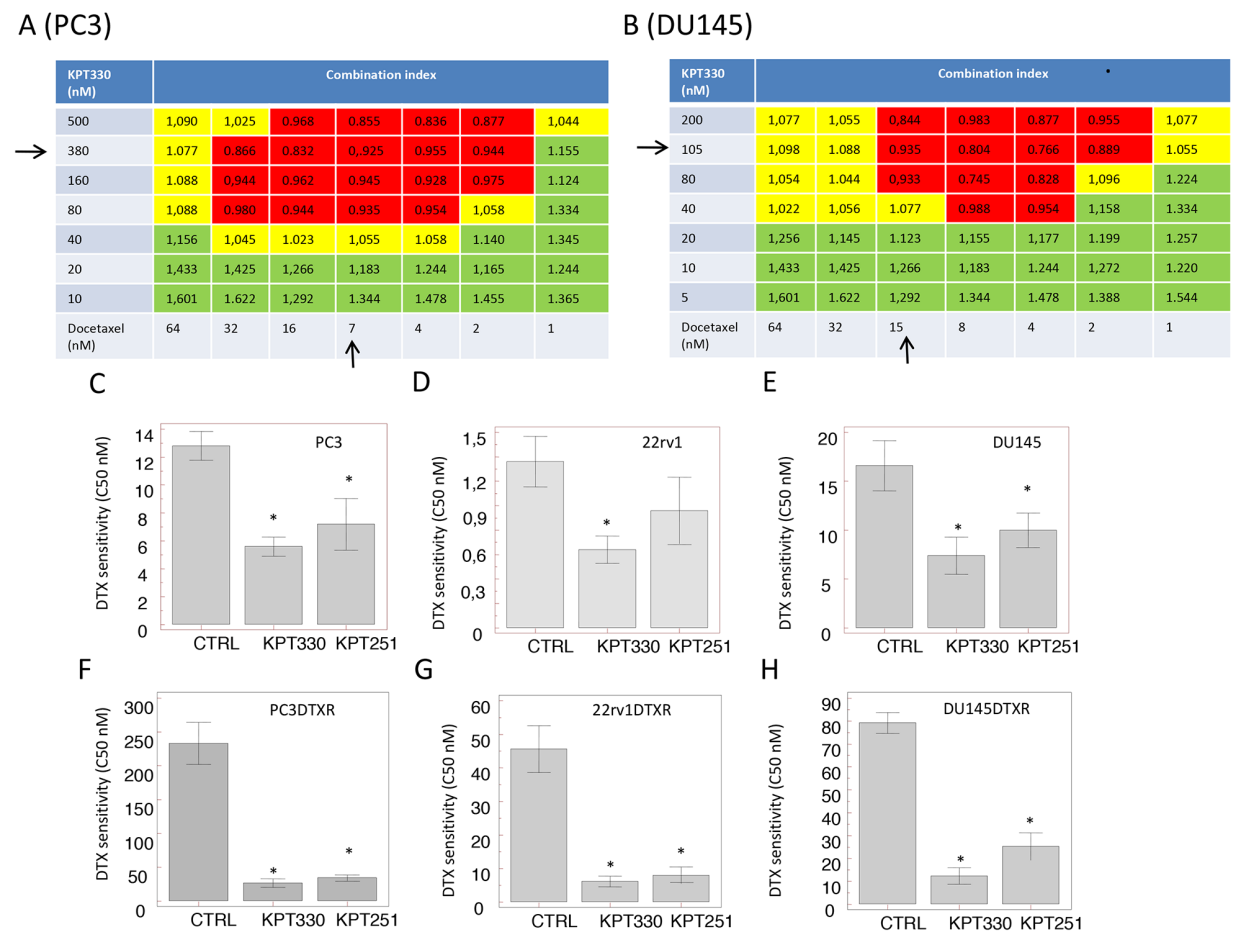
Synergy head maps for Combination Index (CI), calculated by Chou-Talay method **(A)** PC3 synergy map calculated for selinexor and DTX co-treatment. **(B)** DU145 synergy map calculated for selinexor and DTX co-treatment. **(C)** Reduction of DTX sensitivity (IC50) calculated for PC3 cultured with selinexor and KPT-251 at IC20 values of 100 nM and 78 nM, respectively. **(D)** Reduction of DTX sensitivity (IC50) calculated for 22rv1 cultured with selinexor and KPT-251 at IC20 values of 75 nM and 56 nM, respectively. **(E)** Reduction of DTX sensitivity (IC50) calculated for DU145 cultured with selinexor and KPT-251 at IC20 values of 50 nM and 58 nM, respectively. **(F)** Reduction of DTX sensitivity (IC50) calculated for PC3DTXR cultured with selinexor and KPT-251 at IC20 values of 120 nM and 85 nM, respectively. **(G)** Reduction of DTX sensitivity (IC50) calculated for 22rv1DTXR cultured with selinexor and KPT-251 at IC20 values of 90 nM and 75 nM, respectively. **(H)** Reduction of DTX sensitivity (IC50) calculated for DU145DTXR cultured with selinexor and KPT-251 at IC20 values of 135 nM and 92 nM, respectively. Synergy is showed in the red boxes, additivity in yellow boxes and competition in green boxes. Black arrows represent the IC50 values for both compounds.

In Figure 5 we show the PC3 (A) and DU145 (B) synergy heat map for combination indices (CIs) calculated by Chou-Talay method. This Figure shows that the combination is synergistic at IC_20_ values for both compounds with more points of synergy observed in PC3 cells than in DU145 cells. In order to calculate the changes of IC_50_ values for DTX in our cell systems we used the IC_20_ values of selinexor or KPT-251 for single cell lines and growing doses of DTX. (Figure 5C–5E) confirms that administration of SINE compounds sensitizes tumor cells to DTX sensitively reducing IC_50_ values for this anticancer agent.

SINE compounds have significant synergistic effects in docetaxel-resistant prostate cancer cell derivatives restoring DTX sensitivity (Figure 5F–5H): co-administration with 50 nM selinexor corresponding to IC_20_ value for PC3DTXR reduced IC_50_ value calculated for docetaxel from 225 nM to 47 nM, a 5-fold reduction. Similarly, co-administration of 125 nM KPT-251 (IC_20_ value [23]) reduced the IC_50_ of DTX 2-fold in the same cells (IC_50_ = 3.7 nM). The decrement observed in 22rv1 was of about 10-fold (44 nM vs 7 nM for selinexor and 6.2 nM for KPT-251) whereas the reduction of IC_50_ in DU145 DTXR was about 6 fold (78 nM vs 12 nM for selinexor and 25 nM for KPT-251).

### XPO1 inhibitors increased DNA damage when used with DTX

**Figure 6:**
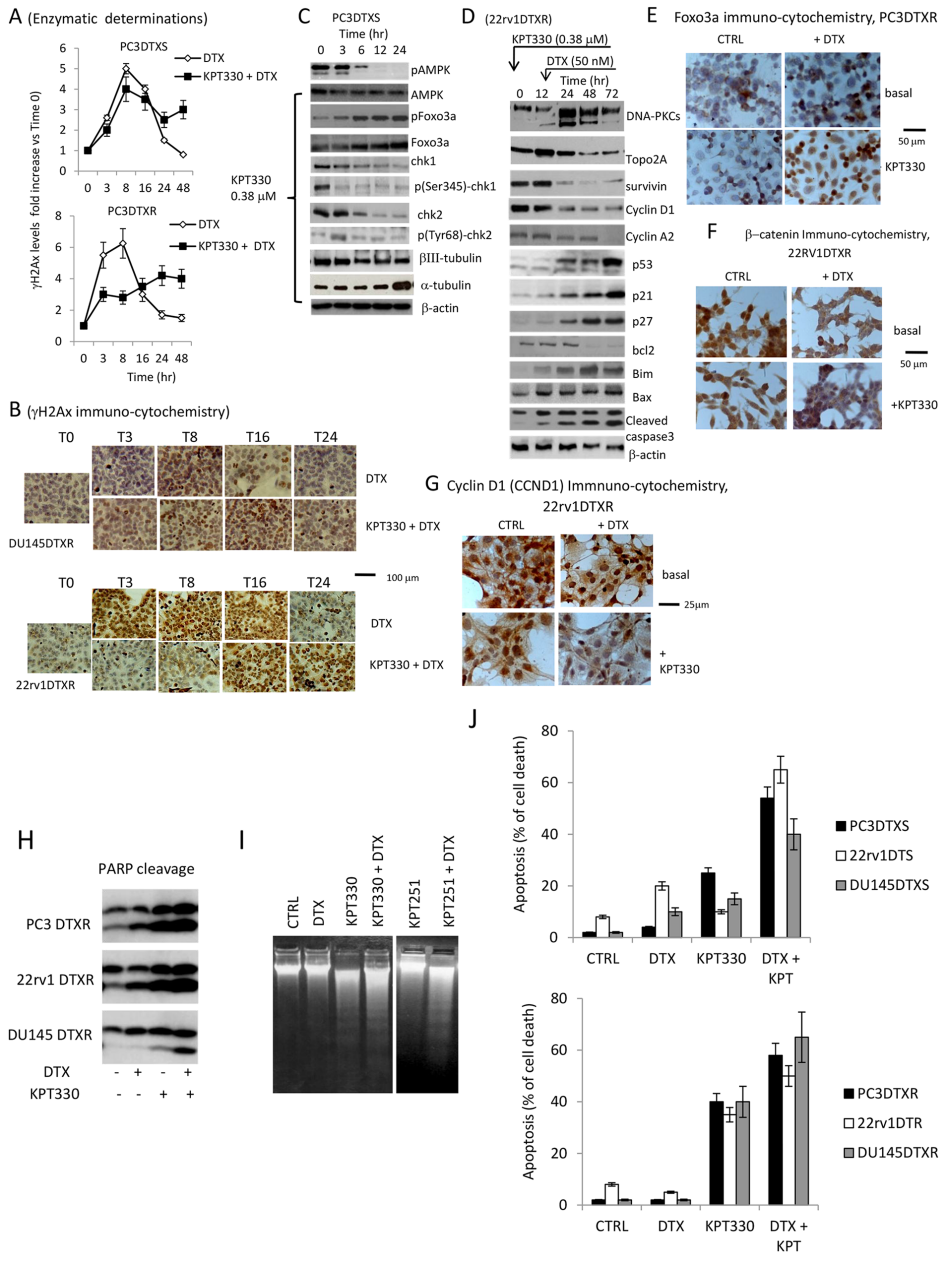
XPO1 inhibitors increased DNA damage when used with docetaxel **(A)** Enzymatic determination of γH2AX histone expression in PC3DTXS and PC3DTXR (a Time course experiment). γH2AX expression levels were considered as fold increase vs Time 0 (T0). **(B)** immunocytochemical eveluation of gH2AX expression in DU145DTXR and 22rv1DTXR cells treated with 80 nM and 40 nM DTX, respectively. **(C)** Western blot analysis for the expression of DNA repair signaling proteins (ATM/pATM; Foxo3a/pFoxo3a; Chk1/p-Chk1 and Chk2/p-Chk2) and bIII tubulin in PC3DTXS cells treated with 380 nM selinexor. **(D)** Combination treatment: western blot analysis for the expression of markers for cell cycle (p21, p27, cyclin D1 and cyclin A2) and apoptosis (bcl2, bim, bax). Induction of caspase 3 activation/cleavage. **(E)** Immunocytochemical evaluation for Foxo3a expression and cytological localization in PC3DTXR cells in CTRL, KPT330 (380 nM), DTX (200 nM) and combination showing increase of Foxo3a in the nucleus of DTX plus Selinexor treated cells. **(F)** Immunocytochemical evaluation for β-catenin expression and cytological localization in 22rv1DTXR cells in CTRL, KPT330 (380 nM), DTX (200 nM) and combination showing a sensitive decrease of β-catenin in the nucleus of DTX plus Selinexor treated cells. **(G)** Immunocytochemical evaluation for c-myc expression and cytological localization in 22rv1DTXR cells in CTRL, KPT330 (380 nM), DTX (200 nM) and combination showing a sensitive decrease of c-myc in the nucleus of DTX plus Selinexor treated cells. **(H)** PARP cleavage in single and combined treatments indicating increased apoptosis and unrepaired DNA after co-administration of KPT330 and DTX. **(I)** DNA ladder confirming increased DNA fragmentation in the combined treatment. **(J)** Apoptosis evaluation by FACS in DTXS and DTXR cells. Each lane was loaded with 100 μg of proteins.

In order to demonstrate that selinexor increased DNA damage and slowed down DNA repair when used in combination with DTX, we evaluated the changes in the γH2AX expression by ELISA in PC3 DTXS and PC3 DTXR cells. This analysis levels revealed that DTX-induced DNA damage signals (such as γH2AX) could not promote complete DNA damage repair in both DTX sensitive and resistant cells after pre-treatment with selinexor (Figure 6A). Indeed, we observed, a complete return to baseline levels of γH2AX at 24 and 48 hours in single agent DTX treatment but nor when DTX was combined with selinexor. In Figure 6B we show immunocytochemical pictures performed on DU145 DTXR and 22rv1 DTXR cell strains demonstrating that in DTXR treated DU145 and 22rv1 cells γH2AX staining was significantly increased up to time 8 (T8) and later. When we considered combined treatments, γH2AX levels reached maximal values at T8-T16 in DU145DTXR cells and at T16-T24 in 22rv1DTXR. Some mitosis of culture treated with DTX and selinexor in combination are strongly γH2AX positive. These mitosis result also aberrant. Next we evaluated the expression changes in Foxo3a, Chk1 and Chk2 after KPT330 (380 nM) administration focusing our attention on PC3 DTXS cells (Figure 6C). We observed that 380 nM selinexor increased steady state levels of FOXO3 and this could be due to p-AMPK down-modulation. This effect was time dependent and maximal from 12-24 hours (Figure 6C). Selinexor was able also to reduce total and phosphorylated levels of Chk1/2 as well as reduce βIII tubulin in the presence of significant levels of α-tubulin, the major target of DTX. Combination treatments were able to modulate several proteins involved in the regulation of cell cycle (p21, p27, cyclin D1 and cyclin A2) and apoptosis (bcl2, bim, bax) inducing caspase 3 cleavage (Figure 6D). In Figure 6E we show immunocytochemical evaluation of the increase of Foxo3a after DTX and combined DTX plus selinexor treatment. Foxo3a appears to be increased mainly in the nucleus of DTX plus selinexor treated cells. Similarly we observed by immuno-cytochemistry that total and nuclear β-catenin (22rv1DTXR, Figure 6F) expression levels were significantly reduced in single DTX or selinexor administration but mainly in combined treatments. Cyclin D1 (DU145DTXR, Figure 6G) levels were increased in the nucleus of DTX treated cells but abrogated after selinexor administration alone or in combination with DTX. In addition, we observed that DTX-induced PARP1 cleavage was increased after combination with selinexor (Figure 6H) as result of a reduced or slowed down selinexor-inhibited DNA damage repair, as previously reported (reference: Ranganathan et al 2016). This finding is associated with increased DNA fragmentation (DNA laddering, Figure 6I) and apoptosis (Figure 6J). In Figure 6H we show also the effects of KPT251.

### *In vivo* anti-tumor effect of SINE compounds in combination with DTX

To determine the effects of selinexor or KPT-251 administration on DTX sensitivity *in vivo* we evaluated two SINE compounds (selinexor and KPT-251) in combination with DTX in PC3, DU145, 22rv1 cell lines, and in DTX resistant PC3 DTXR. The cells were subcutaneously injected in athymic male nude mice. In order to reduce the probability of biases due to differences in tumor engraftment we analyzed the tumor progression the parameter “Time to Progression (TTP)”, defined as the time (days) necessary to double the tumor volume for each tumor, comparing differences of TTP by Kaplan Meyer distribution. Xenografted mice were randomly assigned to receive therapeutic doses of selinexor, KPT-251 or DTX and combinations as described in Materials and methods.

We demonstrate that combination between selinexor and DTX (Tables 1 and 2) significantly increased the efficacy of single treatments evaluated by tumor weight reductions measured at the end of drug administration in PC3, DU145 and 22rv1. Selinexor restored also the sensitivity to DTX of PC3 DTXR (Table 2). The calculation of combination indices revealed that the combination involving selinexor and DTX significantly increased the efficacy of single treatments evaluated as tumor weight reductions with synergistic effects both in PC3 DTXR (CI=0.64) and 22rv1 (CI=0.50) xenografts and additive effects in PC3 (CI=0.95) and DU145 (CI=1.12) xenografts. The number of tumors in which progression was: (i) 10/10 in the animal groups of CTRL and in those treated with selinexor, KPT-251 and DTX, and 7/10 (selinexor + DTX) and 8/10 (KPT-251 + DTX) in PC3 tumors; (ii) 10/10 in the groups of CTRL and in those treated with DTX, selinexor, KPT-251 and in the combination KPT-251 + DTX and 6/12 in the group treated with selinexor + DTX in DU145 tumors; (iii) 10/10 in the groups of CTRL and in those treated with DTX, selinexor and KPT251, whereas progression was observed in 6/10 in the group of animals treated with selinexor + DTX and 8/10 in that treated with KPT-251 and DTX in 22rv1 tumors.

**Table 1:** Antitumor activity of DTX alone or in combination with KPT330 or KPT251 in PC3 and 22rv1 xenografts

Cell line	Drug	Weight of mice (gr +/- SE)	Tumor weight (mg +/- SE)	TTP (days +/_ SE)	Vessel count (+/- SE)	Ki67 (% +/- SE)	Apoptosis (% +/- SE)
PC3	Vehicle	25,8 ± 0.6	880 ± 140	9.0 ± 1,0	23.2± 2.3	55.5 ± 3.5	< 2
	DTX	26.0 ± 0.4	627 ± 172	13.8 ± 1,5	11.5 ± 0.5	34.5 ± 2.5	8.5 ± 1.0
	KPT-330	23.8 ± 0.5	560± 94	14.4 ± 0.5	6.4 ± 0.5	25.0 ± 1.0	5.0 ± 0.5
	KPT-251	24.5 ± 0.3	666± 94	12.2 ± 0.6	12.0 ± 0.5	30.0 ± 2.0	2.5 ± 0.5
	DTX + KPT-330	24.5 ± 0.3	279 ± 59	22.2 ± 0.8	1.3 ± 0.5	10.0 ± 1.5	34.5 ± 3.5
	DTX + KPT-251	24.8± 0.6	355± 41	18.9 ± 0.7	4.7 ± 0.5	20.0 ± 1.0	17.0 ± 1.5
22rv1	Vehicle	26.5 ± 0.3	1045 ± 212	10.0 ± 1.5	30.5 ± 2.5	44.0 ± 3.0	< 2
	DTX	24.5 ± 0.4	709 ± 145	13.5 ± 1.0	20.0 ± 2.0	35.5 ± 2.0	10.0 ± 0.5
	KPT-330	24.0 ± 0.4	527 ± 72	17.0 ± 1.5	8.0 ± 0.5	24.5 ± 2.5	8.5 ± 1.1
	KPT-251	23.8 ± 0.55	615±55	15.0 ± 1.0	14.5 ± 0.5	32.0 ± 1.5	< 2
	DTX + KPT-330	24.5 ± 0.3	200± 88	22.5 ± 2.0	5.0 ± 0.5	10.5 ± 2.0	44.5 ± 0.5
	DTX + KP-T251	25.5 ± 0.3	279 ± 68	20.5 ± 1.5	10.0 ± 0.5	15.5 ± 1.5	30.5 ± 3.5

**Table 2:** Antitumor activity of DTX alone or in combination with KPT330 or KPT251 in DU145 and PC3DTXR xenografts

Cell line	Drug	Weight of mice (gr +/- SE)	Tumor weight (mg +/- SE)	TTP (days +/_ SE)	Vessel count (+/- SE)	Ki67 (% +/- SE)	Apoptosis (% +/- SE)
DU145	Vehicle	24.5 ± 0.5	798 ± 210	12.6 ± 3.3	24.9 ± 2.4	36.5 ± 3.5	<2
	DTX	25.3 ± 0.5	331 ± 86	17.6 ± 2.1	11.5 ± 1.5	16.4 ± 2.5	8.5 ± 1.5
	KPT-330	23.8 ± 0.5	477 ± 145	16.4 ± 2.6	6.8 ± 1.2	22.0 ± 3.0	4.0 ±1.0
	KPT-251	21.5 ± 1.0	360± 82	15.6 ± 3.2	7.0 ± 0.5	16.5 ± 1.8	7.5 ± 0.5
	DTX + KPT-330	21.5 ± 0.5	178 ± 52	22.6 ± 4.1	4,8 ± 0.2	11.8 ± 2.8	19.5 ± 2.5
	DTX + KPT-251	21.0± 0.5	224 ± 67	21.6 ± 3.5	3.0 ± 0.1	9.5 ± 0.5	24.5 ± 2.0
PC3DTXR	Vehicle	25.0 ± 1.0	1150± 192	8.0 ± 1.0	35.5 ± 3.0	50.0 ± 3.0	< 2
	DTX	25.3 ± 0.5	1227 ± 270	8.5 ± 2.0	38.0 ± 2.0	54.5 ± 2.5	<2
	KPT-330	24.0 ± 0.5	775 ± 110	13.0 ± 1.5	16.0 ± 0.5	28.0 ± 1.5	12.0 ± 2.5
	KPT-251	22.0 ± 1.0	825 ± 120	11.0 ± 1.0	22.0 ± 2.5	35.0 ± 4.0	8.5 ± 1.0
	DTX + KPT-330	22.5 ± 0.5	450 ± 50	17.5 ± 2.0	5,5 ± 0.5	8.0 ± 1.0	38.0 ± 5.0
	DTX + KPT-251	21.0± 1.0	545± 88	15.5 ± 1.5	8.0 ± 0.5	13.0 ± 2.0	21.0 ± 3.0

**Figure 7:**
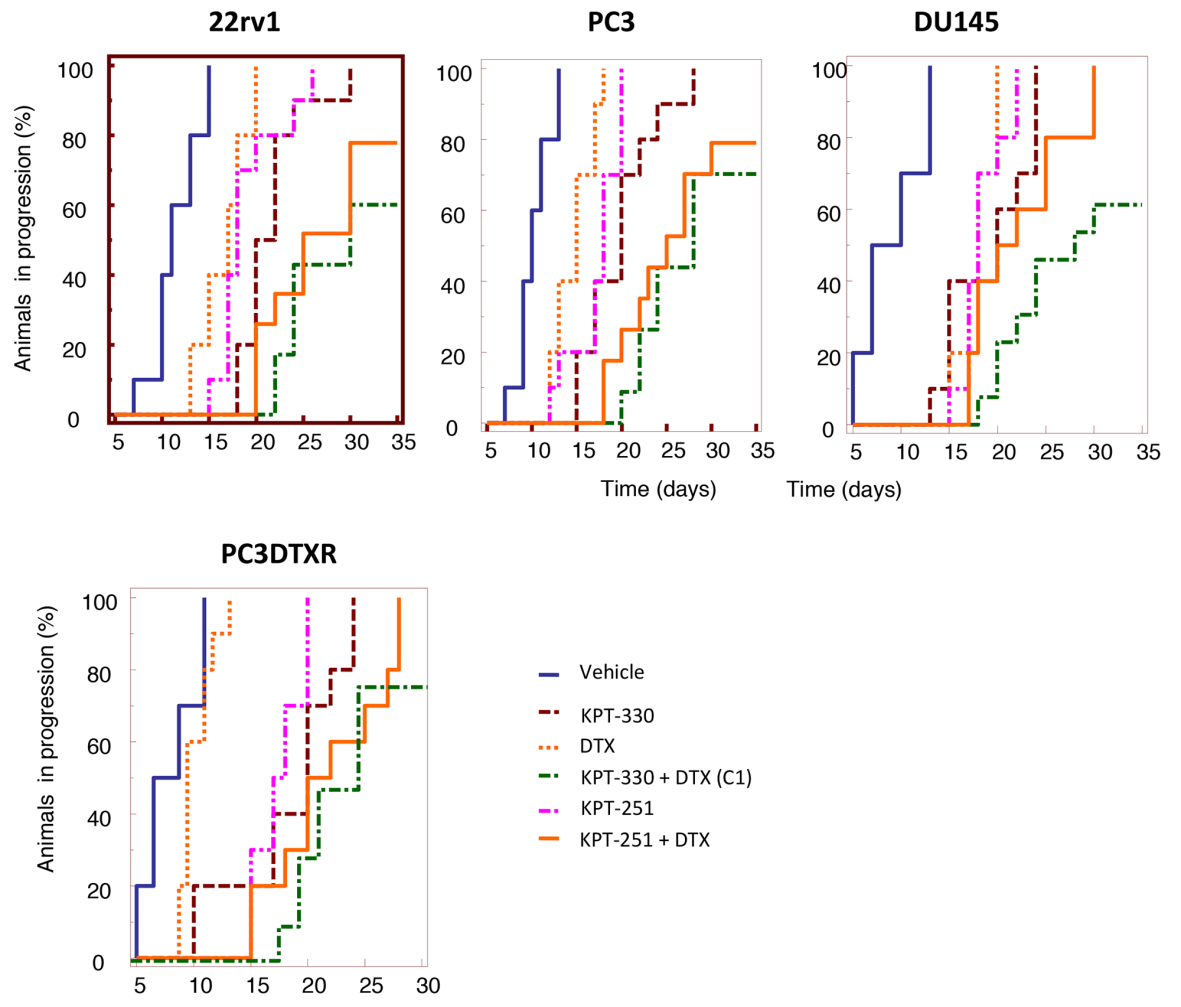
*In vivo* experiments Kaplan-Meier estimates for rates of progression in 22rv1 PC3, DU145 and PC3DTXR subcutaneous tumors.

**Figure 8:**
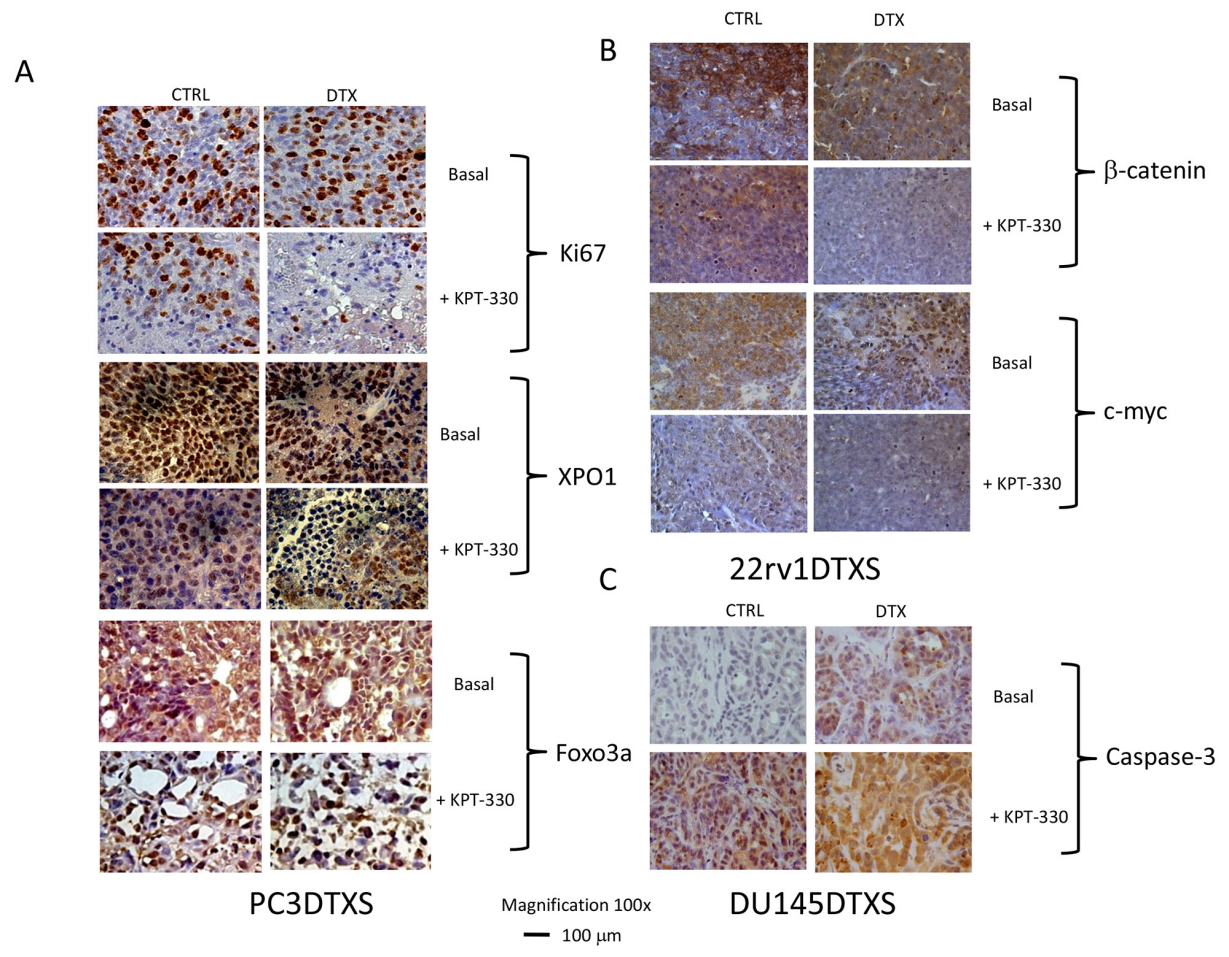
Immunohistochemical analyses of PCa xenograft treated with DTX, KPT330 or combinations *in vivo* **(A)** Evaluation of Ki67, XPO1 and Foxo3a in PC3DTXS xenografts. **(B)** β-catenin and c-myc expression in 22rv1DTXS xenografts. **(C)** Caspase-3 expression in DU145DTXS xenografts.

In Figure 7 we show the Kaplan Meyer graphic representation of the Time to Progression calculated in the single animal groups whereas the calculation of Hazard ratios and statistics were enclosed in the Table 3. To explore cellular mechanisms that could account for the anti-tumor effects we assessed immuno-histochemically the tumor cells proliferation (Ki67); apoptosis (TUNEL and cleaved-caspase 3) and angiogenesis modification (mouse CD31). Data are summarized in Tables 1 and 2 whereas some pictures of treated and untreated tumors are shown in Figure 8).

**Table 3:** Statistical analysis performed on Time to Progression Kaplan Meyer curved generated for DTX sensitive Pca cells and DTX resistant PC3 cell line

	PC3		22rv1		DU145		PC3DTXR	
Comparison	Hazard ratio	Significance	Hazard ratio	Significance	Hazard ratio	Significance	Hazard ratio	Significance
DTX vs vehicle	3.16	P=0.0008	3.36	P=0.0002	6.06	P<0.0001	1.75	P=0.2028 (NS)
KPT330 vs vehicle	7.82	P<0.0001	7.84	P<0.0001	8.49	P<0.0001	5.66	P<0.0001
KPT-330 vs DTX	2.48	P=0.0025	2.33	P=0.0086	1.40	P=0.1954 (NS)	3.24	P<0.0001
C1 vs vehicle	17.96	P<0.0001	18.52	P<0.0001	19.52	P<0.0001	10.71	P<0.0001
C1 vs DTX	5.70	P<0.0001	5.51	P<0.0001	3.22	P<0.0001	6.12	P<0.0001
C1 vs KPT-330	2.30	P<0.0001	2.36	P=0.0055	2.30	P=0.0012	1.89	P=0.0175
KPT-251 vs vehicle	5.37	P<0.0001	5.20	P<0.0001	6.68	P<0.0001	4.62	P<0.0001
KPT-251 vs DTX	1.70	P=0.1846 (NS)	5.51	P<0.0001	1.10	P=0.7665 (NS)	3.24	P<0.0001
C2 vs vehicle	13.24	P<0.0001	12.87	P<0.0001	12.36	P<0.0001	8.33	P<0.0001
C2 vs DTX	4,20	P<0.0001	3.83	P<0.0001	2.04	P=0.0884 (NS)	4.76	P<0.0001
C2 vs KPT-251	2.46	P=0.0036	2.47	P=0.0040	1.85	P=0.1428 (NS)	1.80	P=0.0116 (NS)
C2 vs C1	1.36	P=0.2554 (NS)	1.44	P=0.2120 (NS)	1.58	P=0.2265 (NS)	1.37	P=0.5449 (NS)
KPT-251 vs KPT-330	1.46	P=0.0213 (NS)	1.51	P=0.1877 (NS)	1.27	P=0.7688 (NS)	1.23	P=0.6290 (NS)

Immunohistochemical analyses show that combined treatment shrinks tumors and exposition of blood vessels, significantly decreased the percentage of Ki67 positive cells when compared to controls or single drug treatments suggesting reduced cell proliferation. (Figure 8A, Tables 1 and 2). Increased apoptosis, detected by TACS BlueLabel-based TUNEL kit assay was also observed (Tables 1 and 2) and was higher in combination treatments when compared to single administrations. In particular in DU145 xenografts, selinexor alone decreased Ki67 expression by 68% whereas apoptosis increased by 4 folds and decreased micro vessel density by 64% (Table 2). DTX decreased Ki67 by 66% expression, increased apoptosis by about 2-folds and decreased micro vessel density to about 44%. The combination of selinexor plus DTX increased antiproliferative activity reducing Ki67 expression by 77% and micro vessel density by 88%; whereas, it increased apoptosis by about 4 folds. The same analyses performed on PC3 and 22rv1 shows similar results with different efficacy (Table 1). We also analyzed the drug effects on the PC3 DTXR model (Table 2). These analyses demonstrate that selinexor alone decreased Ki67 by 44% whereas apoptosis increased by 12 times and decreased micro vessel density by 38%. DTX shows similar Ki67 expression, and micro vessel density. The combination of selinexor plus DTX restored the antiproliferative activity, reducing Ki67 expression and micro vessel density by 84% whereas, it increased apoptosis by 3 times. In addition we observed that total expression of XPO1 was also decreased in combined treatments (Figure 8A) as result of the possible DTX-mediated nuclear accumulation (our *in vitro* data, see above) and selinexor-mediated XPO1 degradation. Next we demonstrated increased expression of Foxo3a in xenograft tissue of mice receiving DTX, The localization was both nuclear and cytoplasmatic. Nuclear expression of Foxo3a was increased in selinexor treated tumors whereas a reduced nuclear and cytoplasmatic expression of Foxo3a was observed in the combined treatment as result of a probable increase in Foxo3a degradation. In Figure 8A we show the IHC pictures obtained in PC3DTXS xenografts. A similar behavior was observed for β-catenin and cyclin D1 expression after combination treatment selinexor and DTX due to increased protein degradation as shown in Figure 8B in 22rv1DTXS xenograft. Increased caspase 3 expression was also demonstrated in combined administration respect to those observed in controls and single treatment as shown in Figure 8C in DU145DTXS xenograft. These results indicate the combination had a greater impact on tumor proliferation and apoptosis then single agents.

## DISCUSSION

Paclitaxel (PTX), an alkaloid that targets microtubules, and its synthetic analogues (i.e. docetaxel, DTX) are anticancer drugs validated against several human solid tumors. This family of compounds alters and disrupts mitosis, cell motility, and the cell proliferation. DTX-resistant (DTXR) cancers highlight the rapid onset of multiple cross-resistance and the high percentage of failures even in therapies that involve drug combinations. Indeed, drug resistance is the most important obstacle for treatment of cancer, including CRPC. Several molecular mechanisms have been identified and are related to increased activation of pathways involved in DNA damage repair and cell survival. An important role is played by increased expression and/or activity of multi-drug resistance proteins such as ABCB1 [60, 61]. In our study, we demonstrated the possibility that acquired DTXR may be overcome by a combinatorial therapy including SINE compounds (XPO1 inhibitors) selinexor (KPT-330) or KPT-251, with the DTX. CRPC drug-sensitive cell lines with their drug-resistant strains were used in this study. Functionally, DTX interferes with microtubule assemblage. It has been demonstrated that paclitaxel resistant PC3 cells show decreased expression of acetylated α-tubulin and the cell cycle regulator p21, and increased expression of βIII tubulin, histone deacetylase 6 (HDAC6), and the anti-apoptotic protein Bcl2. In our cell models α-tubulin was lower cells whereas βIII tubulin and HDAC6 were higher in resistant cells when compared with levels observed in parental cells. Similarly, these proteins were modulated during the treatment with DTX in sensitive cells from short term to long term culture (data not shown). DTX-mediated microtubule rearrangement alters cell division resulting in double-strand DNA breaks and subsequent apoptotic cell death. It has been widely demonstrated that metastatic CRPC can have deep genomic aberrations that interfere with DNA repair [62, 63]. In addition, several tumor suppressor proteins (TSP) and growth regulatory proteins (GRP) are mislocalized in cancer cells by overexpression and activity of XPO-1 [15–31]. Nuclear and cytoplasmic expression of XPO-1 is elevated in prostate tumors when compared to normal and hyperplastic tissue [37]. Here we demonstrate that XPO-1 is overexpressed in DTX resistant CRPC cells and differentially localize into the cytoplasm where, several proteins targeted by this cargo, are overexpressed. Some of these proteins (i.e. survivin) are able to reduce apoptosis whereas others increase drug efflux. SINE compounds, which inhibit XPO1 activity, have been demonstrated to have anticancer effects in models of PCa [23, 37]. In this report, we further demonstrate that XPO-1 is a potential target for the treatment of aggressive CRPC cells in association with DTX. DTX sensitive and DTX Resistant cells were also considered for the evaluation of *in vitro* effects. When XPO-1 inhibitors were used in combination with DTX, DNA damage signaling was increased (increased phospho-H2AX (ϒH2AX) levels), but the rate of DNA repair was significantly reduced since ϒH2AX levels were maintained for prolonged times when compared to DTX alone. This results in increased apoptosis and improves the effectiveness of DTX. This two-drug combination was also highly effective against DTX-resistant CRPC cells. Reduction in tumor growth was dose-dependent and associated with inhibition of cellular proliferation and activation of apoptosis, which correlated with PARP and caspase-3 cleavage. SINE compounds are potent therapeutic tools to treat aggressive/castration resistant PCa cells. This appears to be due to the modulation of a multiple signaling pathways including time-dependent cyclin D1 and survivin decreases in expression. It has been demonstrated that cyclin D1 knockdown/decrement reduces cell proliferation and increases sensitivity to chemotherapy [64] and when sequestered in the cytoplasm [65] induces apoptosis. It has been observed that selinexor and KPT-251 reduces the export of cyclin D1 and P21WAF1 from the nucleus and reduce overall expression of cyclin D1 following prolonged exposure. Abnormalities in the regulation of cyclin D1 expression [66] and cell-cycle inhibitory genes (p21WAF1, p16INK4a, and p27KIP1) have been reported in PCa [67]. P21WAF1 mainly localizes to the cytoplasm where it play an anti-apoptotic role. However, when localized in the nucleus, P21WAF1 prevents cell cycle progression at the G1 phase. Similarly nuclear localization of P27KIP1 enables this regulatory function.

High levels of survivin expression are independent risk factors for poor prognosis in several cancers and the cytoplasmic localization of survivin is particularly high in prostate tumors [68], whereas increased nuclear expression of survivin is a favorable prognostic factor [68]. These observations suggest that nuclear survivin is suppressive for tumor growth and further targeting the cytoplasmic, antiapoptotic fraction of survivin would be an ideal therapeutic avenue. Treatment with selinexor or KPT-251 initially promotes survivin nuclear localization, but at later time points leads to a reduction in its protein levels, which correlates with the timing of cellular antitumor effects of these compounds. Together, this supports a hypothesis in which XPO-1 inhibition leads to a loss of survivin levels, thus inhibiting tumor growth and enhanced apoptosis. Reduced expression of TUB3, survivin and cyclin D1 conditions tumor cells to be more responsive to DTX. In addition the antitumor and chemosensitizing effects of SINE compounds are sustained by other molecular changes. Foxo3a is overexpressed in DTX resistant cells where PI3K/Akt/mTOR signaling pathways are constitutively activated. In this state, Foxo3a is constitutively inactive being in the cytoplasm. We observed that KPT-251 and selinexor significantly increase nuclear localization of FOXO3a, where it becomes active and this is due also by reduced activation of PI3K/Akt/mTOR signaling pathways with increased nuclear accumulation of Foxo3a. We observed also that knockdown of FOXO3a expression using small interfering RNA attenuated sensitively the docetaxel efficacy (data not shown).

A better understanding of the downstream cellular targets of docetaxel and SINE compounds will provide information on its mechanism of action and the potential of combined treatment in CRPC. Analogous to observed effects in lung cancer following cisplatin treatment, nuclear accumulation of FOXO3a can be an important player for SINE-dependent DTX sensitization in DTXR PCa cells. The molecular mechanisms regulating FOXO3a cellular localization, however, are complex and largely unknown. XPO-1 plays a key role in the shuttling proteins, including Foxo3a, from the nucleus to the cytoplasm. The phosphorylation of FOXO3a on the Ser7, S253 or Tyr32 residues can lead to the nuclear localization of FOXO3a. Ser7 phosphorylation-deficient mutants seem to be still able to activate the expression of Foxo3a target genes [69]. In contrast, S253 and Thr32 [70] have been shown to play a significant role in the nuclear translocation of Foxo3a. It has also been shown that the inhibition of FOXO3a phosphorylation at Thr32, observed after Akt inhibition, induces FOXO3a nuclear accumulation in lung cancer cells with increased expression of the FOXO3a-dependent apoptotic protein Bim. DTX and SINE compounds induce Foxo3a nuclear accumulation and the activity of apoptotic signaling proteins. Moreover, this class of compounds has been used in combination studies with standard chemotherapies, for example with Topoisomerase II and Proteasome Inhibitors [71]. Altogether, this data suggests that SINE compounds, in combination with DTX, could be used as therapeutic tools for advanced/castration resistant prostate tumors.

## MATERIALS AND METHODS

### Reagents and drug preparation

All materials for tissue culture were purchased from Hyclone (Cramlington, NE, USA). Plasticware was obtained from Nunc (Roskilde, Denmark). Antibodies including: H3 histone [FL-136, sc-10809], c-myc [9E10, sc-40], β-catenin [H-102, sc-7199] p-GSK3β Ser9 [sc11757], GSK3β [H76, sc-9166], XPO-1 [sc-5595], p53 [sc126], p-Akt Ser473 [sc-135651], p-Akt Thr308 [sc135650], PARP-1 [H-250, sc-7150], DNA-PKCs [H-163, sc-9051], Chk1 [G-4, sc-8408], Chk2 [A-12, sc-5278], Topoisomerase 2A [T22C5, sc-65743], ABCB1 known as Mdr-1 [D-11, sc-55510], HDAC6 [H-300, sc-11420], βIII tubulin [TU-20, sc-51670], α-tubulin [B-7, sc-5286] and acetylated α-tubulin were purchased from Santa Cruz (SantaCruz, CA, USA). Antibodies targeting Foxo3a [ab37409], p-FOXO3A [ab31109], p(S345)-Chk1 [ab47318] and p(Thr68-Chk2 [ab32148] were purchased from Abcam (Cambridge UK). Antibodies against Cyclin D1 [2878], AMPKα [5832], phospho-AMPKα (Thr172) [2535] were purchased from Cell Signaling (Danvers, MA, USA). The Ki67 antibody (clone MIB-1) was purchased from Dako (Dako Italia, Cernusco sul Naviglio [MI], Italy). Tunel assay kit [S71003] was purchased from Merck KGaA (Darmstadt, Germany). Survivin antibody was purchased from Biorbyt.

SINE compounds (KPT-251 and selinexor) were provided by Karyopharm Therapeutics Inc., Newton, MA whereas DTX was purchased from Selleck Chemicals (Aurogene, Rome, Italy). For *in vitro* studies, all compounds were dissolved in DMSO and stored at -20°C until use

### Cell lines

Three commercial (22rv1, DU145 and PC3) models for CRPC were purchased from DSMZ and ATCC. PC3, 22rv1 and DU145 DTX-resistant cells (DTXR) were selected by cultivating PCa cells in the presence of 10 nM DTX as previously described [51, 52]. Selected clones were pooled and maintained for under continuous 10 nM docetaxel exposure followed by incremental doses of DTX for at least six months. To minimize the risk of misidentified and/or contaminated cell lines, DNA profiling was periodically carried out in-house to authenticate cell cultures. DNA was isolated from cell lines using a standard DNA isolation kit. STR profiling was performed using GenePrint® 10 System (Promega Corporation, Madison, WI). An eight-capillary 3500 Genetic Analyzer (Applied Biosystems Life Technologies Europe BV, Monza, Italy) was used to separate and identify alleles using standard procedures. GenePrint® 10 System allows co-amplification and detection of eight human loci required by the guidelines ASN-0002. For non-commercial cell lines, the authentication process was carried out by comparing STR-fingerprints with those published by Adri van Bokhoven and co-workers [53]. In addition, cell lines were stocked at very low passages and used at <15-20 subcultures.

### Growth assays

Cells were seeded at a density of 2 x 10^4^ cells/mL in 24-well plates. Cells were left to attach and grow in 5% FCS DMEM for 24 h. After this time, cells were maintained in the appropriate culture conditions. Morphological controls were performed every day with an inverted phase-contrast photomicroscope (Nikon Diaphot, Tokyo, Japan). Cells were trypsinized and resuspended in 1.0 ml of saline, then counted using a NucleoCounter™ NC-100 (automated cell counter systems, Chemotec, Gydevang, Denmark). The effect on cell proliferation was measured by taking the mean cell number with respect to controls over time for the different treatment groups. The half-maximal inhibitory concentration (IC_50_; concentration of drug required for a 50% reduction in growth/viability) values and combination index (CI) values of the SINE molecules when used alone and in combination with other drugs were determined by using the Dojindo Cell Counting Kit-8 (Dojindo EU GmbH, Munich, Germany).

For drug combination experiments, cell viability assays were performed as described above, and the results were analyzed for synergistic, additive, and antagonistic effects using the CI method developed by Chou and Talalay [54].

### Cell viability and apoptosis assay

Viable cells were counted using the NucleoCounter™ NC-100 (automated cell counter systems, Chemotec, Cydevang, DK). Apoptosis was evaluated by using Tali® Apoptosis Kit - Annexin V Alexa Fluor® 488 & Propidium Iodide-based, (Life Technologies Italia, Monza, Italy). Stained cells were then measured on a Tali® Image-Based Cytometer. Apoptosis was further confirmed by FACS analysis following the instructions of the manufacturer.

### Western blot

Cytoplasmic and nuclear protein extracts were obtained by using the Nuclear/Cytosol Fractionation Kit from Biovision Inc. (Milpitas, CA, USA). Cell extracts and conditioned media from treated and untreated cells were electrophoresed under reducing conditions and transferred to nitrocellulose filter (Schleicher and Schuell GmbH, Dassel, Germany). Reactive bioluminescent bands were visualized by using the detection kit (Supersignal, Perbio Science, Tattenhall, UK) using Bio-Rad gel Doc™ (Bio-Rad Laboratories S.r.l., Milan, Italy).

### Xenograft model

Male CD1 nude mice (Charles River, Milan, Italy) were maintained under the guidelines established by the University of L’Aquila, Medical School and Science and Technology School Board Regulations. Experiments on animals have been approved by your local IRB in compliance with the Italian government regulation n.116 January 27, 1992 for the use of laboratory animals which is line with ARRIVE guidelines. All mice received subcutaneous flank injections of 1 x 10^6^ PC3, DU145, 22v1 cells or PC3DTXR. Tumor growth was measured bi-weekly with a Vernier caliper (length x width). Tumor weight was calculated according to the formula: TW (mg) = tumor volume (mm^3^) = d2 x D/2, where d and D are the shortest and longest diameters, respectively. The effects of the treatments were examined as previously described [55, 56]. Animals were sacrificed by carbon dioxide inhalation and tumors were subsequently removed surgically. A piece of tumor was frozen in liquid nitrogen for protein analysis and another piece was fixed in paraformaldehyde overnight for immunohistochemical analyses.

### Treatments

Mice were treated by oral gavage with either vehicle control (Pluronic F-68/PVP-K29/ 32), selinexor or KPT-251. Groups of 10 animals were considered. Before tumor injection, animals were randomized to receive (i) vehicle (PBS 1 mL/kg i.p., 2/week and povidone/pluronic F68 1 mL/kg p.o., 3/week), (ii) selinexor (10 mg/Kg, q 2d × 3 weeks, po), (iii) docetaxel (i.p. injection of 7.5 mg/kg per week), (iv) selinexor (110 mg/Kg, q 2d × 3 weeks, po) + DTX (i.p. injection of 7.5 mg/kg per week). KPT-251, was also tested at 30 mg/kg q2d × 3 weeks, po and in the same combinations. Treatments were started when tumor volumes reached approximately 80 mm^3^ (Day 0) and were stopped after 28 days. The following parameters were used to quantify the antitumor effects upon different treatments: (1) tumor volume measured during and at the end of experiments, (2) tumor weight measured at the end of experiment, (3) complete response (CR) defined as the disappearance of the target lesion with respect to baseline, (4) tumor progression (TP) defined as an increase of greater than 50% of tumor volume with respect to baseline, (5) time to progression (TTP). *In vivo*, combinational studies were evaluated by CalcuSyn (Biosoft). For the calculation of CI, the values of cell kill for a fixed tumor volume were considered (determined by the log cell kill (LCK)). LCK was determined as LCKZ (TKC)/(3.3KTd), where Td represents the mean control group doubling time required to reach a fixed tumor volume, expressed in days, whereas T and C are the same values as described above [55].

### Immunohistochemical analyses

Indirect immunoperoxidase staining of tumor xenografts samples was performed on paraffin embedded tissue sections (4 μm). Briefly, sections were incubated with primary antibodies overnight at 4°C. Next, avidin–biotin assays were done using the Vectastain Elite kit obtained from Vector Laboratories. Mayer’s hematoxylin was used as nuclear counterstain. Ki67 labeling index was determined by counting 500 cells at 100X and determining the percentage of cells staining positively for Ki67. Apoptosis was determined by using the TACS TdT in situ TACS Blue Label kit (code 4811-30-K; R&D Systems, Inc. Minneapolis, MN). Apoptosis was measured as the percentage of tunnel positive cells +/- SD measured on five random fields (400 X).

Tumor microvessels were counted at ×400 in five arbitrarily selected fields and the data were presented as number of CD31+ microvessels / ×100 microscopic field for each group. The presence of red cells in tumor tissue and in blood vessels as well as the presence of micro-thrombi and bleeding zones was demonstrated by Martius yellow-brilliant crystal scarlet blue technique. Tumor hemoglobin levels were quantified as described elsewhere [41].

### Statistical analysis

Continuous variables were summarized as the mean and SD or 95% CI for the mean. Statistical comparisons between controls and treated groups were established by carrying out the ANOVA test or by Student’s t-test for unpaired data (for two comparisons). Dichotomous variables were summarized by absolute and/or relative frequencies. For dichotomous variables, statistical comparisons between control and treated groups were established by carrying out the exact Fisher’s test. For multiple comparisons, the level of significance was corrected by multiplying the P value by the number of comparisons performed (n) according to the Bonferroni correction. Overall survival was determined by Kaplan–Meier analysis and a Gehan’s generalized Wilcoxon test. When more than two survival curves were compared, the Logrank test for trend was used. This tests the probability that there is a trend in survival scores across the groups. All tests were two-sided and were determined by Monte Carlo significance. P values <0.05 were considered statistically significant. In the figures in which statistical analysis was performed, significance is indicated by an asterisk. SPSS (statistical analysis software package, IBM Corp., Armonk, NY, USA) version 10.0 and StatDirect (version. 2.3.3, StatDirect Ltd, Altrincham, Manchester, UK) were used for statistical analysis and graphical presentation.
