# Correction: Pharmacological treatment with inhibitors of nuclear export enhances the antitumor activity of docetaxel in human prostate cancer

**DOI:** 10.18632/oncotarget.27289

**Published:** 2019-10-29

**Authors:** Giovanni Luca Gravina, Andrea Mancini, Alessandro Colapietro, Francesco Marampon, Roberta Sferra, Simona Pompili, Leda Assunta Biordi, Roberto Iorio, Vincenzo Flati, Christian Argueta, Yosef Landesman, Michael Kauffman, Sharon Shacham, Claudio Festuccia

**Affiliations:** ^1^ Department of Biotechnological and Applied Clinical Sciences, Laboratory of Radiobiology, University of L’Aquila, L’Aquila, Italy; ^2^ Department of Biotechnological and Applied Clinical Sciences, Division of Radiotherapy, University of L’Aquila, L’Aquila, Italy; ^3^ Department of Biotechnological and Applied Clinical Sciences, Division of Human Anatomy, University of L’Aquila, L’Aquila, Italy; ^4^ Department of Biotechnological and Applied Clinical Sciences, Division of Molecular Pathology, University of L’Aquila, L’Aquila, Italy; ^5^ Department of Biotechnological and Applied Clinical Sciences, Division of Applied Biology, University of L’Aquila, L’Aquila, Italy; ^6^ Karyopharm Therapeutics, Newton, MA, USA

**This article has been corrected:** Due to the high number of replicates in the experiments reported in Figures 1A and 2A, an erroneous selection of some individual well images was made during the assembly of these two panels. This problem occurred due to the pattern similarity between the erroneously selected images and the correct pictures. The corrected Figure 1 and Figure 2 are shown below. The authors declare that these corrections do not change the results or conclusions of this paper.

Original article: Oncotarget. 2017; 8:111225–111245. 111225-111245. https://doi.org/10.18632/oncotarget.22760

**Figure 1:**
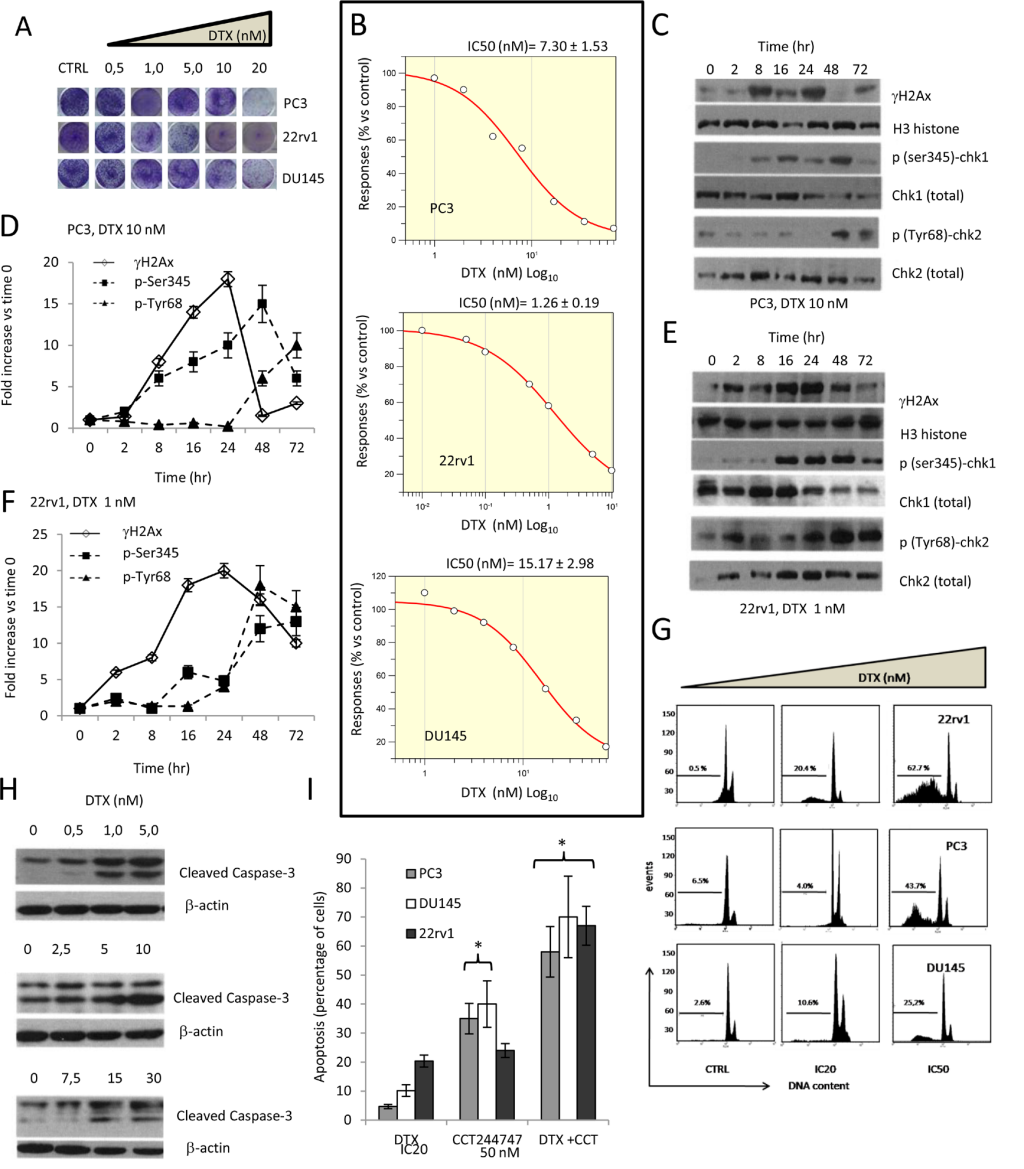
Effects of docetaxel (DTX) in DTX sensitive (DTXS) PC3, 22rv1 and DU145 cell lines. (**A**) Plate image representation of crystal violet stained PC3, 22rv1 and DU145 cells cultured in 24 well/plates with different doses of DTX (0-20 nM). (**B**) Proliferation curve generated with Graftit software for PC3 (IC50=7.21 nM), 22rv1 (IC50=1.26 nM) and for DU145 cells (IC50=15.17 nM). (**C**) DTX (10.0 nM) induces time-dependent DNA damage response (DDR) characterized by γ-H2AX phosphorylation and Chk1/2 kinase activation in PC3 cells. (**D**) The levels of γH2AX, Chk1 and Chk2 were normalized by using H3 histone as housekeeping nuclear protein, whereas those of phosphorylated forms of Chk1 (p-Ser345) and Chk2 (p-Tyr68) with total expression of Chk1 and chl2, respectively. Normalizing expression levels were plotted in the time for PC3 cells. (**E**) DTX (1.0 nM) induces time-dependent DNA damage response (DDR) characterized by γ-H2AX phosphorylation and Chk1/2 kinase activation in 22rv1 cells. (**F**) The levels of γH2AX, Chk1 and Chk2 were normalized by using H3 histone as housekeeping nuclear protein, whereas those of phosphorylated forms of Chk1 (p-Ser345) and Chk2 (p-Tyr68) with total expression of Chk1 and chl2, respectively. Normalizing expression levels were plotted in the time for 22rv1 cells. (**G**) FACS analyses for apoptotic rate in CTRL and DTX treated cells at IC20 and IC50 values. IC20 values were 0.5 nM, 5.8 nM and 10 nM for 22rv1, PC3 and DU145, respectively (**H**) caspase 3 activation /cleavage. (**I**) Addition of 50 nM Chk1 inhibitor CCT244747 start and accelerate the program of cell death in PC3, 22rv1 and DU145 cells. Each lane of western blots was loaded with 100 μg of proteins. Graphical data derived from three different western blot analysis performed on different cell extracts. Data presented as mean ±Error Standard (ES). Data for γH2AX are statistically significant at all considered times whereas p(SER235)-Chk1 and p(Tyr68)-Chk2 levels were significant starting from 16 and 24 hour, respectively (p<0.001).

**Figure 2:**
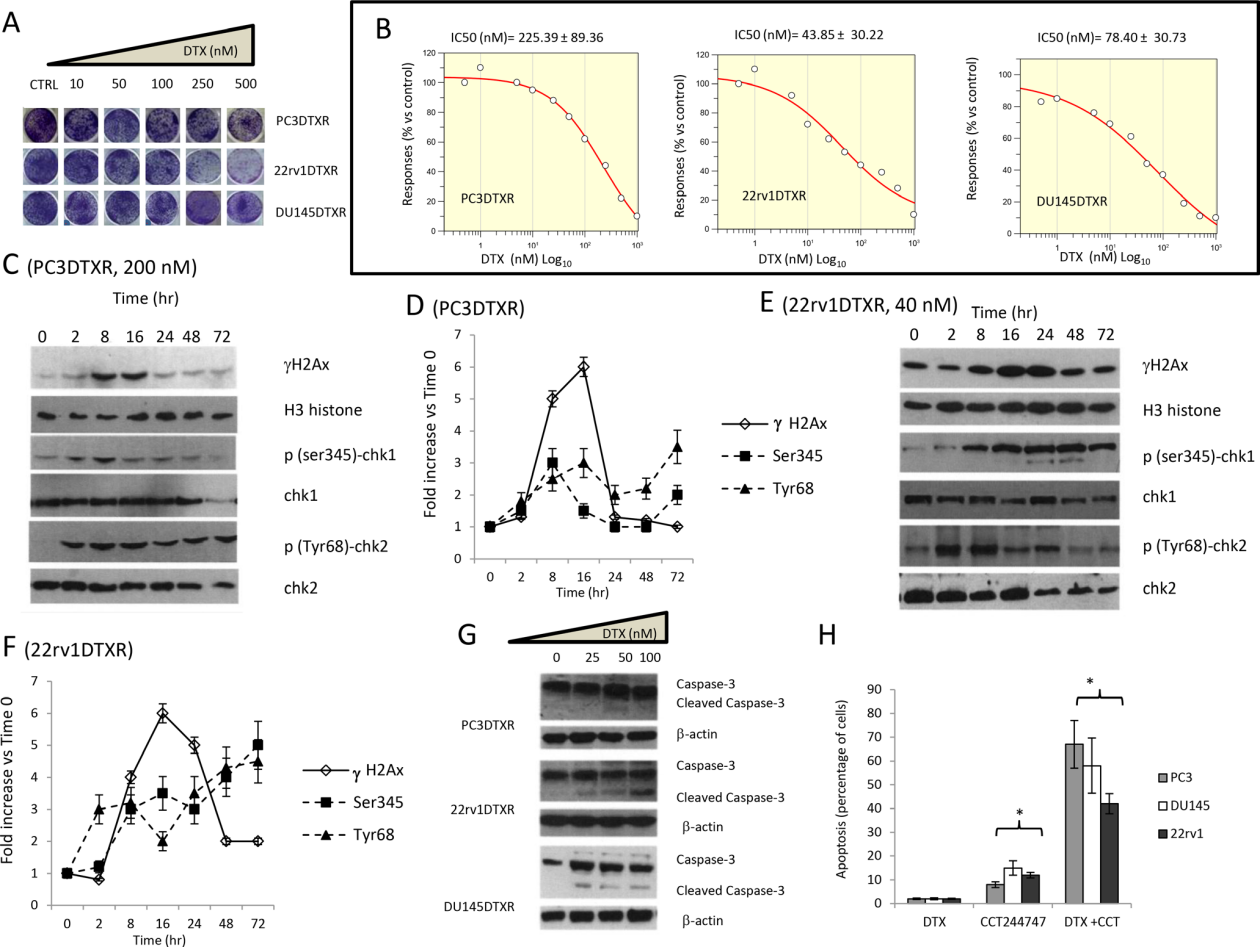
Effects of docetaxel (DTX) in DTX resistant (DTXR) PC3, 22rv1 and DU145 cell lines. (**A**) Plate image representation of crystal violet stained PC3DTXR, 22rv1DTXR and DU145DTXR cells cultured in 24 well/plates with different doses of DTX (0-500 nM). (**B**) Proliferation curve generated with Graftit software for PC3DTXR (IC50=225.39 nM), 22rv1 (IC50=43.85 nM) and for DU145 cells (IC50=78.40 nM). (**C**) DTX (200 nM) induces time-dependent DNA damage response (DDR) characterized by γ-H2AX phosphorylation and Chk1/2 kinase activation in PC3DTXR cells. (**D**) The levels of γH2AX, Chk1 and Chk2 were normalized by using H3 histone as housekeeping nuclear protein, whereas those of phosphorylated forms of Chk1 (p-Ser345) and Chk2 (p-Tyr68) with total expression of Chk1 and chl2, respectively. Normalizing expression levels were plotted in the time for PC3DTXR cells. (**E**) DTX (40 nM) induces time-dependent DNA damage response (DDR) characterized by γ-H2AX phosphorylation and Chk1/2 kinase activation in 22rv1DTXR cells. (**F**) The levels of γH2AX, Chk1 and Chk2 were normalized by using H3 histone as housekeeping nuclear protein, whereas those of phosphorylated forms of Chk1 (p-Ser345) and Chk2 (p-Tyr68) with total expression of Chk1 and chl2, respectively. Normalizing expression levels were plotted in the time for 22rv1DTXR cells. (**G**) caspase 3 activation /cleavage. (**H**) Addition of 50 nM Chk1 inhibitor CCT244747 start and accelerate the program of cell death in PC3DTXR, 22rv1DTXR and DU145DTXR cells. Each lane of western blots was loaded with 100 μg of proteins. Graphical data derived from three different western blot analysis performed on different cell extracts. Data presented as mean ±Error Standard (ES). In PC3DTXR cells, data for γH2AX are statistically significant starting to 2 hours and until 24 hours; p(SER235)-Chk1 was statistically significant at 8 hour and at 72 hour whereas only p(Tyr68)-Chk2 levels significantly followed a time-dependent increase (p<0.001). In 22rv1DTXR γH2AX, p(Ser235)-Chk1 and p(Tyr68)-Chk2 levels followed a statistically significant time-dependent increase (p<0.001).

